# Microbiome and metabolome profiles of high screen time in a cohort of healthy college students

**DOI:** 10.1038/s41598-022-07381-3

**Published:** 2022-03-02

**Authors:** Paniz Jasbi, Alex E. Mohr, Xiaojian Shi, Tara Mahmood, Qiyun Zhu, Meg Bruening, Haiwei Gu, Corrie Whisner

**Affiliations:** 1 College of Health Solutions, Arizona State University, Phoenix, AZ USA; 2 Systems Biology Institute, Yale University, West Haven, CT USA; 3 Department of Nutrition, Texas A&M University, College Station, TX USA; 4 School of Life Sciences, Arizona State University, Tempe, AZ USA; 5 Biodesign Center for Fundamental and Applied Microbiomics, Arizona State University, Tempe, AZ USA; 6 Center for Translational Science, Florida International University, Port St. Lucie, FL USA; 7 Biodesign Institute Center for Health Through Microbiomes, Arizona State University, Tempe, AZ USA

**Keywords:** Risk factors, Metabolomics, Microbiome

## Abstract

As screens are increasingly integrated into every facet of modern life, there is growing concern over the potential effects of high screen time. Previous studies have largely utilized self-report data on mood and behavioral aspects of screen time, and no molecular theory has yet been developed. In this study, we explored the fecal microbiome and metabolome of a diverse group of 60 college students, classified by high (≥ 75 min/day) or low (0–75 min/day) self-reported screen time using 16S rRNA amplicon sequencing, targeted liquid chromatography-tandem mass spectrometry, and targeted detection of short-chain fatty acids using gas chromatography-mass spectrometry. Several key taxa and metabolites were significantly altered between groups and found to be highly co-occurrent. Results of pathway and enzyme enrichment analyses were synthesized to articulate an integrated hypothesis indicating widespread mitochondrial dysfunction and aberrant amino acid metabolism. High screen time was also predicted to be significantly associated with type I diabetes, obesity, chronic fatigue syndrome, and various manifestations of inflammatory bowel. This is the first-ever study to report the effects of high screen time at the molecular level, and these results provide a data-driven hypothesis for future experimental research.

## Introduction

Screen time, or the time spent using a phone or tablet device, computer, television, or game console, is a matter of increasing public and scientific concern^1^. Electronic screen use has expanded as new technologies are increasingly integrated across all domains of life, including work, entertainment, physical activity, education, travel, finance, and even romance^2^. Screen-based activities are particularly prevalent in younger demographics^3^, with data suggesting significant increases in daily average screen time from infancy to adolescence. In 2013, daily average screen time for children under three in the US was estimated to be 42 minutes^4^, while a 2014 survey of the same measure for Australian children and adolescents (8–18 years old) was estimated to be 7 h and 38 minutes^5^. Estimates of screen time for college students are limited and range widely from 2.8 to 11.6 h per day^6^, and show that female students and physically active students report significantly fewer minutes of total screen time than their male and physically inactive counterparts, respectively^7^.

As widespread use of screen-based devices has grown, so have concerns over their possible impact on physical and mental health. However, current research is largely focused on the relationship between screen-based activity and mental/behavioral health issues. For instance, results from longitudinal cohort studies have provided evidence that higher levels of daily screen time may impede childhood development^8^ and have been positively associated with depression during adolescence^9^. Other problematic areas include reductions in sleep quality and physical activity^10^, as well as increased consumption and desire for alcohol and sweets^11^. These findings have spurred recent guidelines on limiting screen time. Stringent limits on screen use for infants and children under five have been expounded by both the World Health Organization (WHO)^12^ and the American Academy of Pediatrics (AAP)^13^: for all children under 5, both the WHO and AAP recommend no more than 60 min of screen time per day. For children and adolescents ages 5 to 18, the AAP recommends regulating the quality of screen content rather than quantity, with specific recommendations related to screen use before bed and limiting the number of screens in the living environment^13^. Although there is no consensus on a safe amount of screen time for adults, associations between well-being and digital technology use show deleterious effects on individuals engaging in more than 120 min of daily screen time^2^.

Far fewer studies have explored risk factors and consequences of screen time on more complex biological data. One study examining consumption of screen-based media in preschool-age children demonstrated significant correlations between higher screen use and measures of white matter tract demyelination^14^. Another study involving magnetic resonance imaging demonstrated a strong, positive association between brain connectivity and time spent reading books whereas a strong, negative association was observed between length of exposure to screen-based media and degree of neuronal arborization^15^. Furthermore, it appears as though physical activity does not compensate for the adverse effects of screen time on the microstructure of the central nervous system^16,17^. No study to date has investigated the potential effects of screen time using a systems biology approach.

Comprised of thousands of different bacterial taxa as well as various archaea, eukaryotic microbes and viruses, the gut microbiota (GM) is now understood to play a significant role in human health^18^. Correspondingly, advances in next-generation sequencing technology have rendered 16S rRNA amplicon sequencing an attractive platform for monitoring changes in the GM^19^ given its high-throughput parallel sequencing, rapid time-to-analysis, and feasibility^20^. As an internal bioreactor, the GM has tremendous functional capacity^21^, producing a broad suite of metabolites that have varying effects on host health^22^. Like the GM, metabolomic profiles also present a unique, person-specific signature and are heavily predicted by host-associated characteristics and environmental factors^23^. Defined as the complete suite of metabolites (small molecules < 2000 Da) present in a biological system, the metabolome can complement microbiomic readouts and is impacted by similar host-associated characteristics^24^. In particular, aqueous metabolites and short-chain fatty acids (SCFAs) play key roles in host-microbe interactions^24^ and bacterial signaling^25^, respectively. For instance, aqueous fecal and plasma metabolites show strong, significant associations to gut microbial diversity^24^ and SCFAs produced by bacterial fermentation of dietary fibers and resistant starch in the colon may enable cell signaling through surface G-protein coupled receptors^25^. Integrating omics-based data is becoming increasingly feasible and offers important biological insight^26^. The GM and associated metabolic output can also be influenced downstream from host behavior including diet, physical activity, and sedentary time^27^.

Previously, Whisner et al. (2018) reported that racially/ethnically diverse first year college students displayed associations of physical activity and screen time with the GM^27^. This population transitions from family-living to independent living conditions where many new behaviors are instilled, while others are eliminated^28^. The GM in adolescence exhibits greater interpersonal variation and lower bacterial diversity compared to adults, which may promote a more malleable biota^29^. During such a formative period, dietary intake, and health behaviors, including physical activity and sedentarism (e.g., screen time), may allow for greater and lasting microbial shifts. Given the increased prevalence of screen time and the critical formalization of the GM in this age group, we aimed to explore potential differences in the fecal microbiome and metabolome by screen time in a subset of the DevilWaste project cohort of college students (*n* = 60)^27^. Specifically, we performed a cut on self-reported screen time, to compare participants by low (0–75 min/day) vs. high (≥ 75 min/day) screen time using 16S rRNA amplicon sequencing in addition to targeted liquid chromatography–tandem mass spectrometry (LC–MS/MS) profiling of aqueous metabolites and targeted gas chromatography (GC)-MS profiling of short-chain fatty acids (SCFAs).

## Results

### Study design and participant characteristics

**Figure 1:**
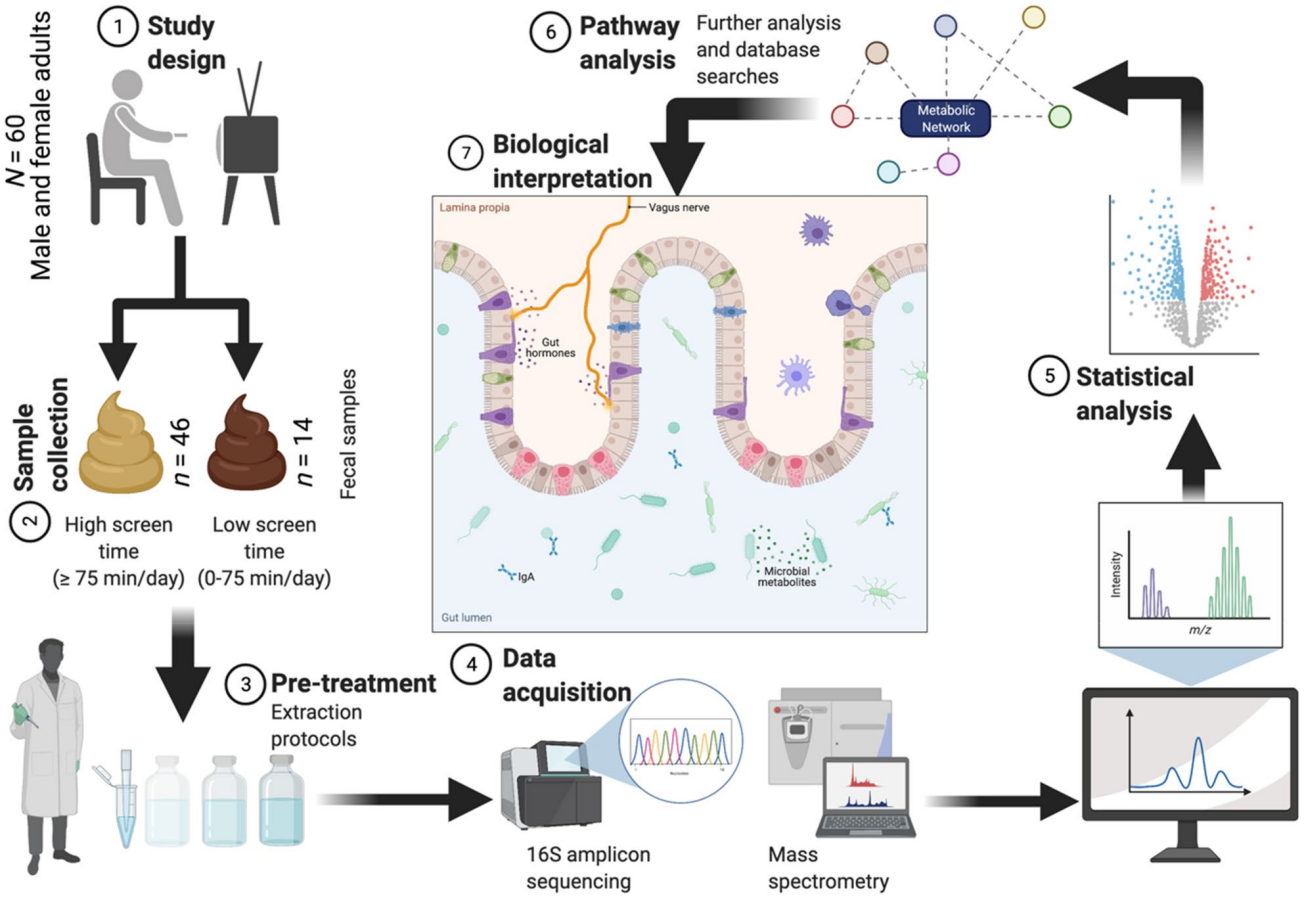
Overview of analytical workflow from study design to biological interpretation. Created with BioRender.com.

The current study is a cross-sectional investigation of metabolomic and microbiomic differences in a cohort of diverse college students; an overview of the analytical workflow is provided in Fig. 1. A total of 60 participants provided a fecal sample and dietary recall, moderate and vigorous physical activity (MVPA), and screen time data (Table 1). The overall mean percentage of kilocalories from carbohydrate, protein, and fat were 46.0 ± 17.9%, 16.9 ± 9.3%, and 37.1 ± 14.7%, respectively (Table 1). Both protein and carbohydrate consumption were within the acceptable macronutrient distribution range (AMDR) of 10–35% and 45–65%, respectively, while the mean fat consumption fell slightly outside the AMDR range of 20–35%. The mean self-reported daily intake of sugar consumed was 75.12 ± 46.13 g/day. Mean daily consumption of dietary fiber for males (*n* = 20) and females (*n* = 40) was 12.21 ± 5.36 g/day and 12.70 ± 5.64 g/day, respectively, which fell below the recommended Adequate Intake for both males (38 g/day) and females (25–26 g/day). No significant difference in self-reported intake (g) of carbohydrates, sugar, fiber, protein, or fat was overserved between high and low screen time groups. Age, sex, body mass index (BMI), and self-reported MVPA did not differ significantly by screen-time classification. Self-reported screen time and MVPA were not significantly correlated (Spearman’s ρ =  − 0.083, *p* = 0.530). An initial principal component analysis (PCA) was performed between high and low screen time groups using the entire set of captured metabolites and GM features (Supplementary Fig. S1). The first two components accounted for approximately 75% of all variance, and analysis of 95% confidence intervals (CIs) showed two potential outliers (DW40 and DW100, one from each study group) which were removed from further analyses upon confirming non-ignorable missingness of metabolomic data.

**Table 1:** Participant characteristics by screen time classification.

	Total (*n* = 60)	Low (*n* = 14)	High (*n* = 46)
Age, mean ± SD	18.5 ± 0.7	18.6 ± 0.9	18.4 ± 0.6
**Sex, % (*n*)**			
Male	33.3 (*20*)	42.9 (*6*)	30.4 (*14*)
Female	66.7 (*40*)	57.2 (*8*)	69.6 (*32*)
**Race/ethnicity, % (*n*)**			
Hispanic	23.3 (*14*)	21.4 (*3*)	23.9 (*11*)
White	43.3 (*26*)	57.1 (*8*)	39.1 (*18*)
Other	33.3 (*20*)	21.4 (*3*)	37.0 (*17*)
**Body Mass Index (kg/m**^**2**^**), mean ± SD**	24.4 ± 5.9	23.3 ± 3.4	24.8 ± 6.5
< 18.5 kg/m^2^% (*n*)	3.3 (*2*)	0 (*0*)	4.3 (*2*)
18.5–24.9 kg/m^2^% (*n*)	63.3 (*38*)	71.4 (*10*)	60.9 (*28*)
25.0–29.9 kg/m^2^% (*n*)	18.3 (*11*)	21.4 (*3*)	17.4 (*8*)
≥ 30.0 kg/m^2^% (*n*)	15.0 (*9*)	7.2 (*1*)	17.4 (*8*)
Screen time (min/day), median (IQR)	195.0 (195.0, 315.0)	15.0 (15.0, 56.6)	195.0 (195.0, 360.0)
Moderate-to-vigorous physical activity (min/day), median (IQR)	52.2 (25.7, 77.1)	58.9 (26.2, 61.6)	45.0 (25.7, 77.1)
**Diet, mean ± SD**	1614.2 ± 589.3	1546.2 ± 703.5	1634.6 ± 559.2
Carbohydrates (g)	186.1 ± 72.5	169.0 ± 61.1	191.3 ± 75.5
Sugar (g)	75.1 ± 46.1	61.6 ± 34.2	79.2 ± 48.8
Fiber (g)	12.6 ± 5.5	11.2 ± 5.5	13.0 ± 5.4
Protein (g)	68.3 ± 37.7	75.2 ± 64.3	66.2 ± 26.0
Fat (g)	66.5 ± 26.5	63.9 ± 33.6	66.4 ± 24.4

*IQR* interquartile range, *SD* standard deviation.

### Diversity metrics did not differ by screen time classification

**Figure 2:**
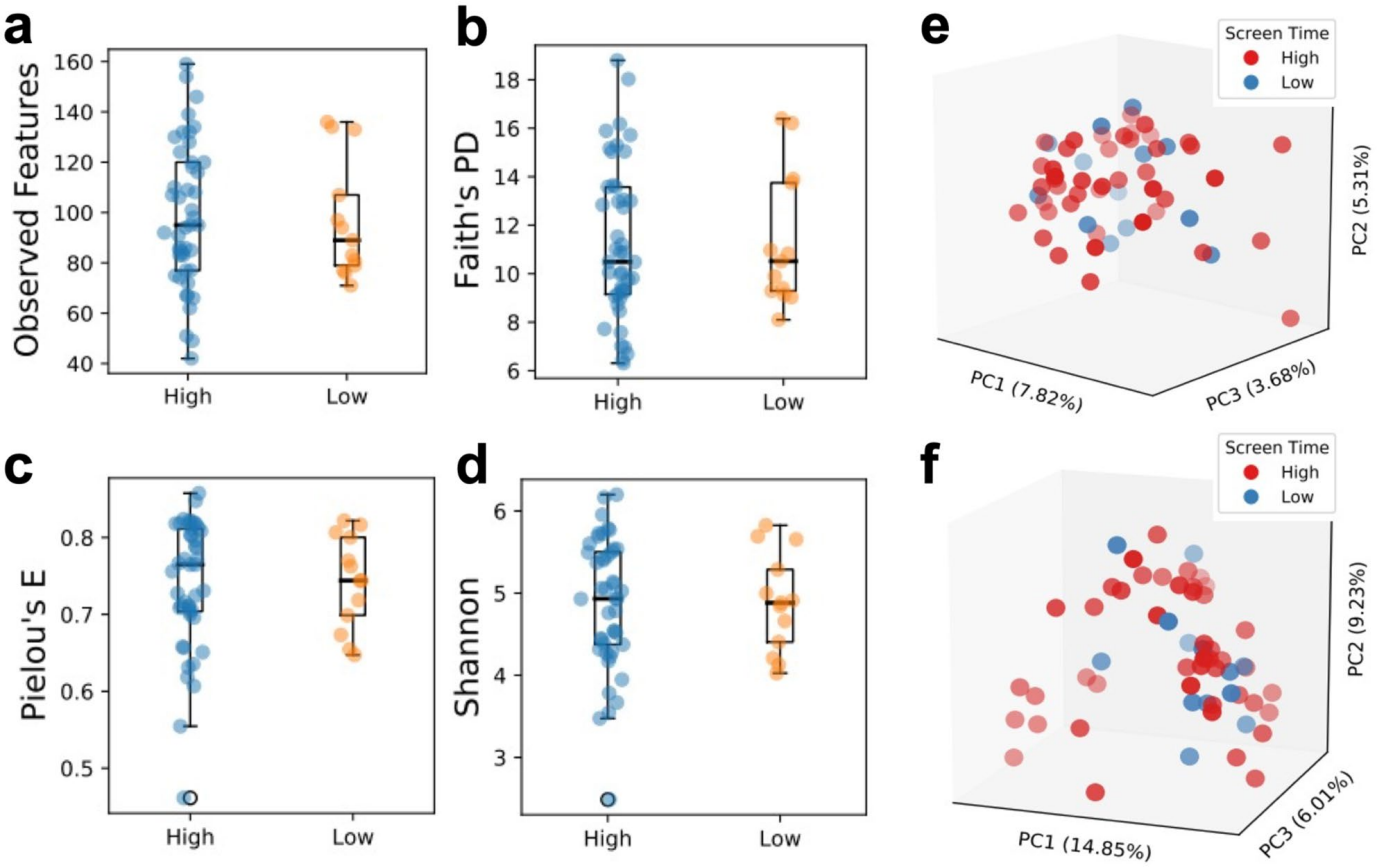
Comparison of alpha and beta diversity metrics between high and low screen time classifications. Boxplots for alpha diversity metrics: (**a**) observed features (unweighted amplicon sequence variants). (**b**) Faith’s PD (phylogenetic diversity). (**c**) Pielou’s E (species evenness). (**d**) Shannon index (weighted proportional abundances). PCoA plots for beta diversity metrics: (**e**) Jaccard. (**f**) Bray–Curtis. Weighted and unweighted UniFrac distance matrices not displayed.

Alpha diversity is an important metric commonly reported in the microbiome literature, and different indices are needed to assess various aspects of within-sample diversity such as richness, evenness, and dominance. Therefore, we have comprehensively reported the most relevant alpha diversity indices in Fig. 2 and Supplementary Table S1 to ensure maximum transparency. When controlling for BMI, age, MVPA, and sex as covariates, no significant differences in alpha diversity measures were observed between high and low screen time groups (observed features, Faith’s PD, Pielou’s E, and Shannon index, *p* ≥ 0.539). These findings suggest that screen time was not associated with GM richness or evenness in this cohort of college students (Supplementary Table S1; Fig. 2a–d). Adonis analysis with the same covariates revealed no significant differences between high and low screen time for beta diversity metrics (Jaccard, Bray Curtis, Unweighted UniFrac, and Weighted UniFrac *p* ≥ 0.219; Supplementary Table S1; Fig. 2e–f).

### Analysis of gut microbiome composition reveals differential abundances in key microbes between groups

**Figure 3:**
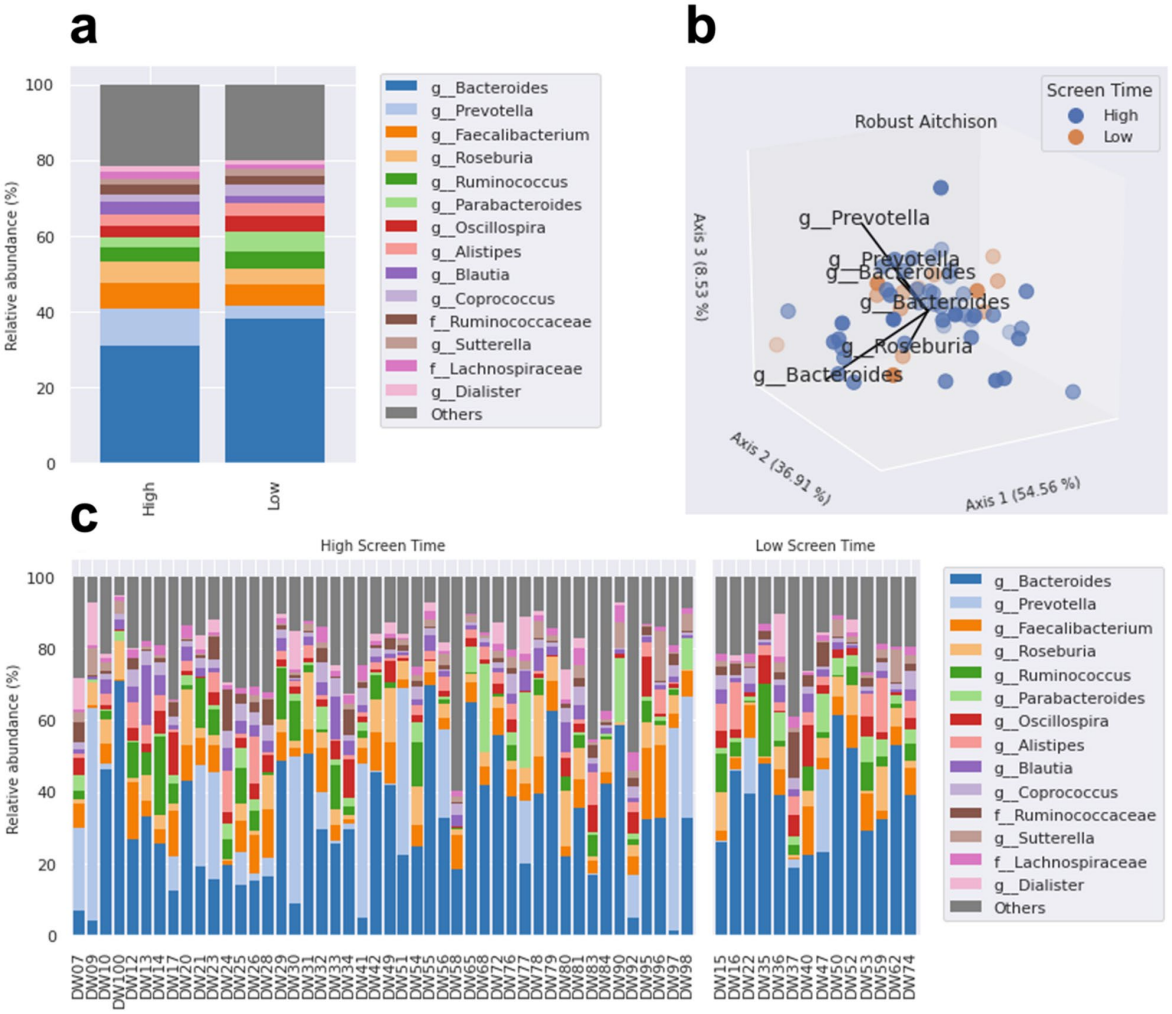
Taxonomic analysis of microbiota between groups. (**a**) Taxonomy bar plot of the top 15 most abundant taxa at the genus level for both groups (*n* = 60). Less abundant taxa, representing ~ 19.5% of relative abundance, are not displayed. (**b**) Robust Aitchison PCA generated from DEICODE showing taxa (arrows) correlated with screen time classification (*n* = 58). (**c**) Taxonomy bar plot of individual subjects at the genus level by high and low screen time groups (*n* = 60). Less abundant taxa are not displayed. Note, where resolution at the genus level was not possible taxa are described at the lowest feature level obtained (i.e., f = family).

After quality control and filtering of any samples with fewer features than our rarefaction threshold (10,000) and low abundance/low prevalence amplicon sequence variants (ASVs) using the qiime2-feature-table plugin with the filter-samples method, we generated and provided taxonomy assignments for 247 features at the genus level (kingdom: 2; phylum: 23; class: 44; order: 69; family: 120; genus: 247). A heatmap of the core microbiome showing prevalence by detection threshold (relative abundance %) at the genus level is shown in Supplementary Fig. S2. Of the top 15 most prevalent genera, we noted several biologically relevant taxa including, *Bacteroides*, *Prevotella*, *Faecalibacterium*, *Roseburia*, *Alistipes*, and *Akkermansia* (Fig. 3a). To reveal salient inter-community niche feature importance based on composition, we used DEICODE, a form of Aitchison distance that is robust to high levels of sparsity^30^. The output is visualized as a robust Aitchison PCA (Fig. 3b), based on the feature table. Upon performing an Adonis test with BMI, age, MVPA, and sex as covariates, no significance was detected between high and low screen time groups (*F* = 0.391, *R*^2^ = 0.008, *p* = 0.678), though we did identify several taxa that were correlated with screen time classification including *Bacteroides*, *Prevotella*, and *Roseburia*. A taxonomy bar plot of individual subjects at the genus level by high and low screen time is provided as Fig. 3c.

**Figure 4:**
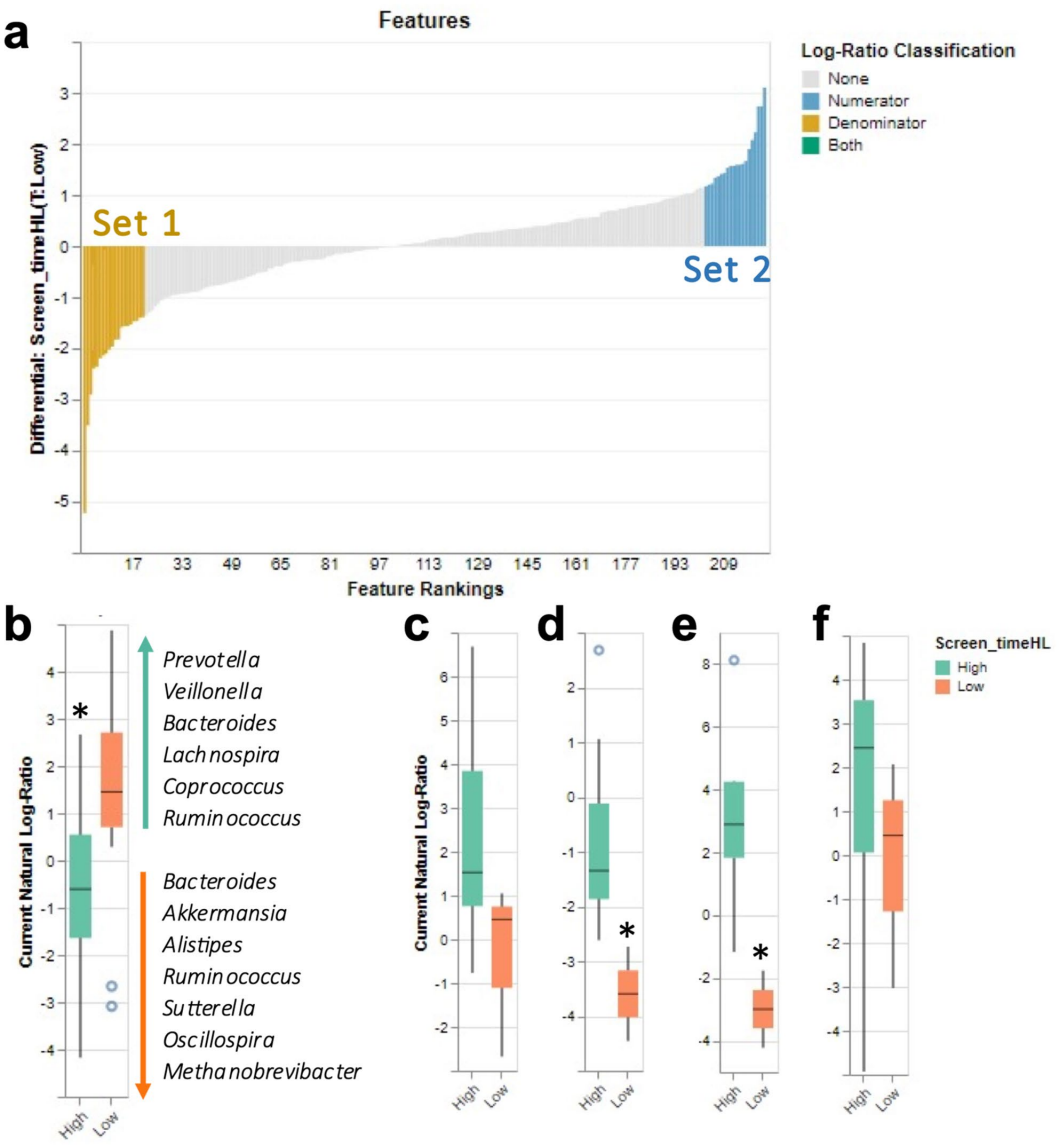
Differential analysis of log ratio classification. (**a**) Comparison of log ratio of the 20 highest and 20 lowest ranked ASVs associated with screen time classification. (**b**) High screen time classification had a significantly greater log ratio of set 2 compared to set 1 (*t*-test); (**c**) *Prevotella//Runimococcaceae* (Mann–Whitney *U* test); (**d**) *Peptostreptococcaceae/Runimococcaceae* (Mann–Whitney *U* test); (**e**) *Bacteroides/Akkermansia* (Mann–Whitney *U* test); (**f**) *Prevotella/Bacteroides* (Mann–Whitney *U* test). *p < 0.05.

To better identify specific microbes associated with screen time classification, we used Songbird, a compositionally aware differential abundance method which provides rankings of features based on their log fold change while accounting for relevant covariates^31^. As before, we controlled for BMI, age, MVPA, and sex as covariates and selected the 20 highest (“set 1,” Supplementary Table S2)- and 20 lowest (“set 2,” Supplementary Table S2)-ranked ASVs associated with screen time classification. Next, we used Qurro^32^ to compute the log ratio of these sets of taxa (Fig. 4a). Comparing the ratios of taxa in this way mitigates bias from the unknown total microbial load in each sample and taking the log of this ratio gives equal weight to relative increases and decreases of taxa^31^. Evaluation of the Songbird model for high/low screen time classification against a baseline model showed exponential decay and a stable plateau, though a *Q*^2^ value of − 0.10 was produced, suggesting a potential for overfitting related to the differences between classifications. Comparing the log ratio of the two sets, we noted the high screen time group had a significantly greater log ratio of set 2 compared to set 1 (Fig. 4b; *p* = 0.002, Cohen’s *d* = 1.89), suggesting that high screen time was more strongly associated with the genera *Prevotella*, *Veillonella*, *Bacteroides*, *Lachnospira*, *Coprococcus*, and *Ruminococcus*. In contrast, low screen time was more associated with the genera *Bacteroides*, *Akkermansia*, *Alistipes*, *Ruminococcus*, *Sutterella*, *Oscillospira*, and *Methanobrevibacter*. To compare these microbial abundances in a compositionally coherent way, we calculated a log ratio with abundances of several high screen time-specific taxa in the numerator and *Ruminococcaceae* (genus level not defined) abundance in the denominator, which Songbird multinomial regression identified as the taxa most associated with low screen time (Fig. 4c–f). Most of these comparisons were not significant, although the high screen time classification showed a significantly greater ratio of *Peptostreptococcaceae/Runimococcaceae* compared to low screen time (*p* = 0.036). High screen time also showed a significantly greater ratio of *Bacteroides/Akkermansia* (*p* = 0.044), but not *Prevotella/Bacteroides* (*p* = 0.518), compared to low screen time.

### Predicted functional profile of the gut microbiome differs by screen time

**Figure 5:**
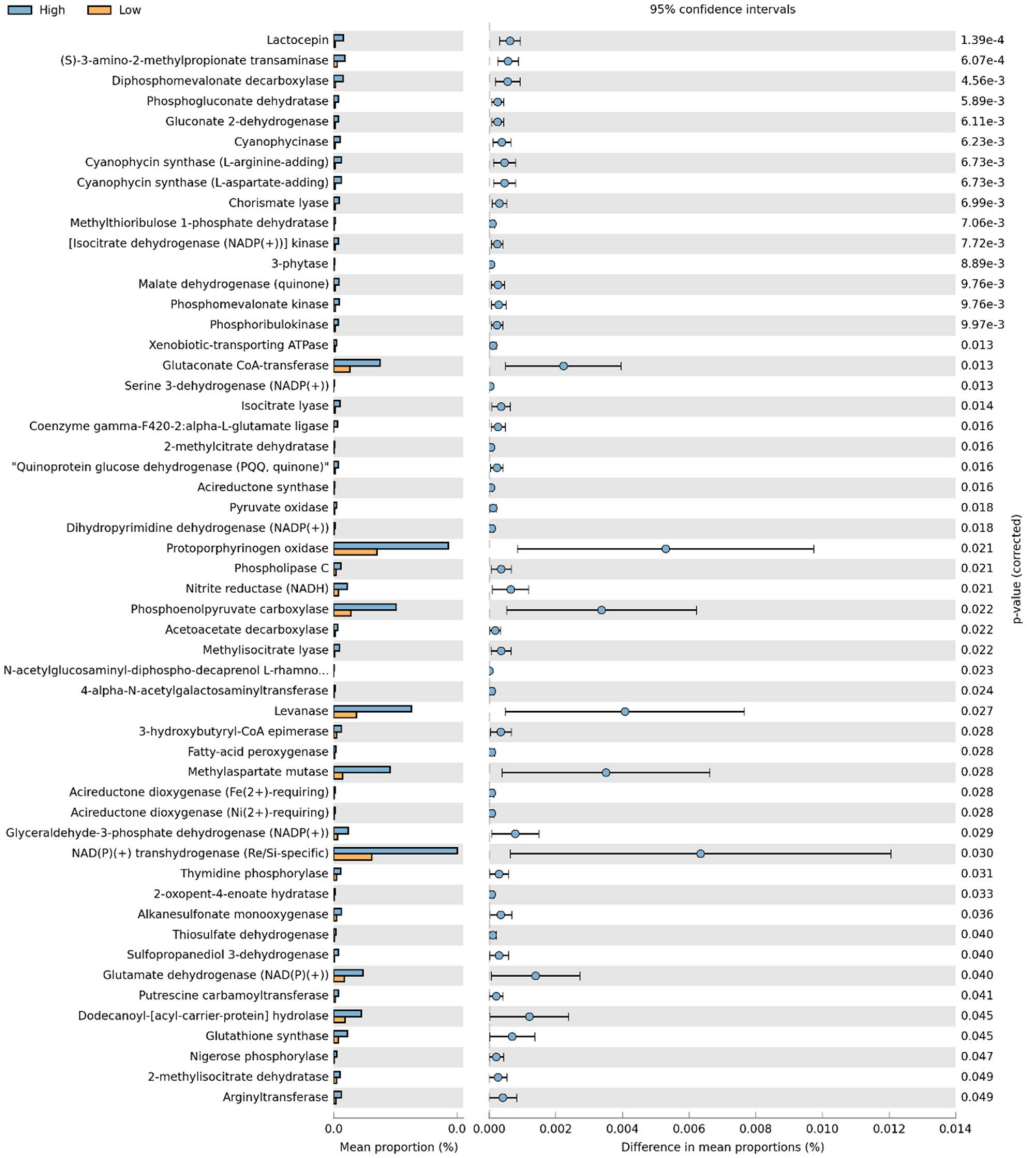
Significantly altered predicted functional KEGG enzymes at level 3 between high and low screen time groups. Single features displayed with *q* value, effect size, and 95% CI (Welch’s *t*-test, *q* < 0.05). Corrected *p* values (*q*) were calculated using Storey's FDR approach.

The Phylogenetic Investigation of Communities by Reconstruction of Unobserved States 2 (PICRUSt 2) pipeline^33^ was used to infer the functional profile of the GM based on 16S amplicon sequencing data. The Kyoto Encyclopedia of Genes and Genomes (KEGG)^34^ outputs were analyzed and illustrated with statistical analysis of the taxonomic and functional profiles. PCA revealed appreciable differences in the predicted functional composition of the GM among the two screen time classifications (e.g., variances accounted by PC 1 and PC 2 were 39.0% and 12.5%, respectively) (Supplementary Fig. S3a). A convergence summary of the predicted metabolic pathways also showed some differential enrichment between the two classifications (Supplementary Fig. S3b). Overall, there were 52 enzymes at KEGG level 3 with significant differences in enrichment between low and high screen time classification (Fig. 5). The main differential enzymes between high and low screen time included glutaconate CoA-transferase (*q* = 0.013), protoporphyrinogen oxidase (*q* = 0.021), phosphoenolpyruvate carboxylase (*q* = 0.022), levanase (*q* = 0.027), methylaspartate mutase (*q* = 0.028), NADP + transhydrogenase (*q* = 0.030), glutamate dehydrogenase (*q* = 0.040), and dodecanoyl-ACP hydrolase (*q* = 0.045).

Taxon set enrichment analysis (TSEA) was also performed using MicrobiomeAnalyst^35^. Predicted functional profiles of host-intrinsic factors such as disease were analyzed using 239 taxon sets, whereas host-extrinsic factors such as diet and lifestyle were predicted using 118 taxon sets. A network view of the least absolute shrinkage and selective operator (LASSO) results is provided in Supplementary Fig. S4. Compared to low screen time classification, subjects in the high screen time group had significantly greater abundance of taxon sets related to Crohn’s disease (*q* = 0.001), type I diabetes (*q* = 0.003), having an overweight/obese mother (*q* = 0.003), and myocardial infarction (*q* = 0.008); high screen time users also had significantly greater abundance of taxa related to consumption of red wine (*q* = 0.007) and coffee (*q* = 0.025). Conversely, the low screen time group had significantly greater abundance of taxon sets related to liver cirrhosis (*q* = 0.001), autism (*q* = 0.006), and high-fat diet (*q* = 0.047), as compared to the high screen time group.

### Analysis of metabolomics data reveals significant differences in five metabolites between high and low screen time users

In total, 140 metabolites were reliably detected from human fecal samples using LC–MS/MS and GC–MS (quality control (QC) CV < 20%, relative abundance > 1,000 in 80% of samples). Relative levels of these 140 metabolites had a median coefficient of variation (CV) of 11.5%, and ~ 76% of captured metabolites had QC CV < 15% (Supplementary Fig. S5). These metabolites spanned 20 different chemical classes and were representative of more than 35 metabolic pathways of potential biological relevance. All metabolomics data were log-transformed and Pareto scaled to approximate normality prior to analysis.

**Figure 6:**
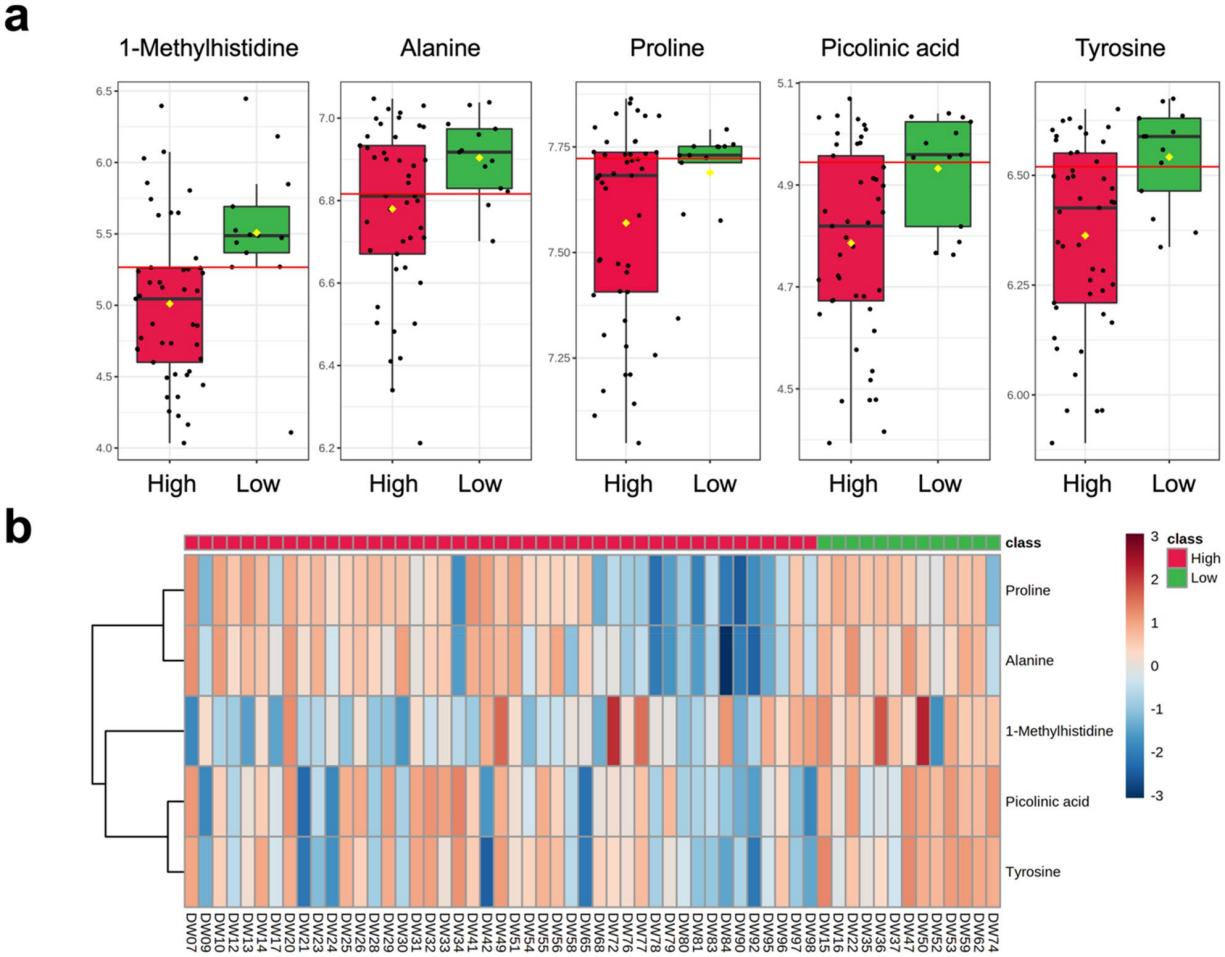
Significant metabolites between high and low screen time groups as determined by GLM with FDR correction. (**a**) Box plots of significant metabolites between groups showing normalized relative abundance. Age, sex, BMI, and MVPA were controlled for as covariates: 1-Methylhistidine (*q* = 0.002), Alanine (*q* = 0.020), Proline (*q* = 0.024), Picolinic acid (*q* = 0.034), Tyrosine (*q* = 0.048). Yellow diamonds signify group means, while horizontal red lines signify optimal between-group cut-offs. (**b**) Heatmap of significant metabolites by screen time classification.

A general linear model (GLM) was used to assess differences in metabolite abundance between high and low screen time groups. Age, sex, BMI, and MVPA were controlled for as covariates, and significance was calculated with FDR correction. Results of the GLM showed five metabolites to be significantly decreased in the high screen time group: 1-methylhistidine (*q* = 0.002), alanine (*q* = 0.020), proline (*q* = 0.024), picolinic acid (*q* = 0.034), and tyrosine (*q* = 0.048) (Fig. 6a). A heatmap of significant metabolites by screen time classification is given in Fig. 6b. Full results of the GLM for all 140 reliably detected metabolites (including mean difference, standard error, FDR *q*, and 95% confidence intervals), are displayed in Supplementary Table S3.

### Predicted functional profile of the fecal metabolome differs by screen time

Enzyme enrichment analysis with LASSO regression was performed using 912 metabolic sets predicted to change in the case of dysfunctional enzymes (Supplementary Fig. S6). Seventeen enzymes were predicted to be significantly increased in the high screen time group (enrichment ratio = 8.0, *p* = 0.003). Two more enzymes, enolase and pyruvate kinase, were also predicted to be significantly increased in the high screen time group (enrichment ratio = 6.0, *p* = 0.020), while another three enzymes were significantly increased in high screen time by an enrichment ratio of 4.0: extracellular chitinase (*p* = 0.040), N-acetyl-D-glucosamine exchange (*p* = 0.040), and peroxisomal FAD transporter (*p* = 0.045). Supplementary Table S4 lists the complete set of enriched enzymes, along with their enrichment ratios and *p* values.

**Figure 7:**
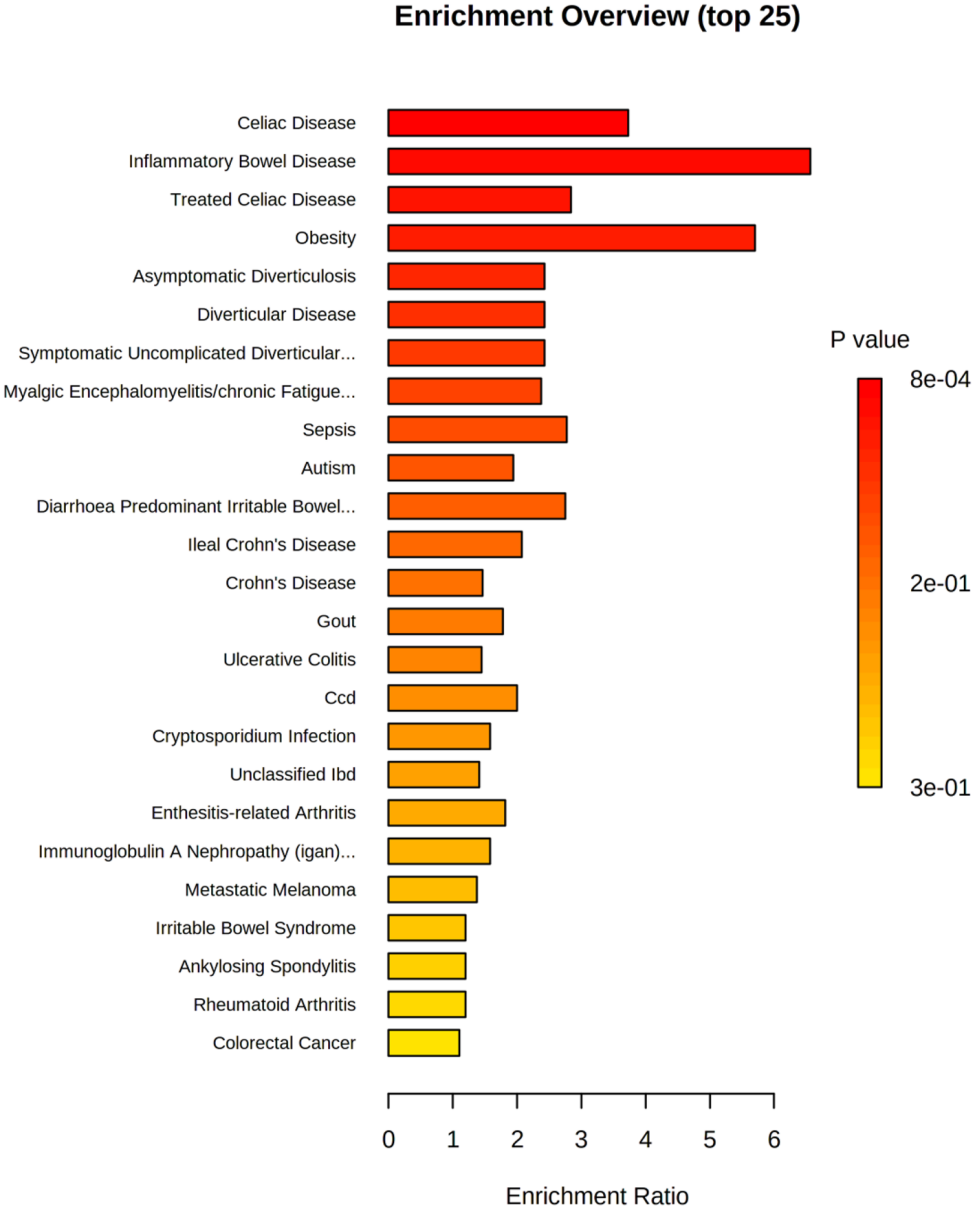
Enrichment analysis of disease signatures performed using 44 metabolite sets reported in human feces. Significant metabolite sets (*p*): celiac disease (< 0.001), inflammatory bowel disease (0.001), treated celiac disease (0.008), obesity (0.016), asymptomatic diverticulitis (0.031), diverticular disease (0.031), symptomatic uncomplicated diverticular disease (0.031), and chronic fatigue syndrome (0.043).

Metabolomics data was also used to perform enrichment analysis of disease signatures using 44 metabolite sets reported in human feces (Fig. 7). Metabolites associated with eight disease signatures were significantly increased in the high screen time group: celiac disease (*p* < 0.001), inflammatory bowel disease (*p* = 0.001), treated celiac disease (*p* = 0.008), obesity (*p* = 0.016), asymptomatic diverticulitis (*p* = 0.031), diverticular disease (*p* = 0.031), symptomatic uncomplicated diverticular disease (*p* = 0.031), and chronic fatigue syndrome (*p* = 0.043).

**Figure 8:**
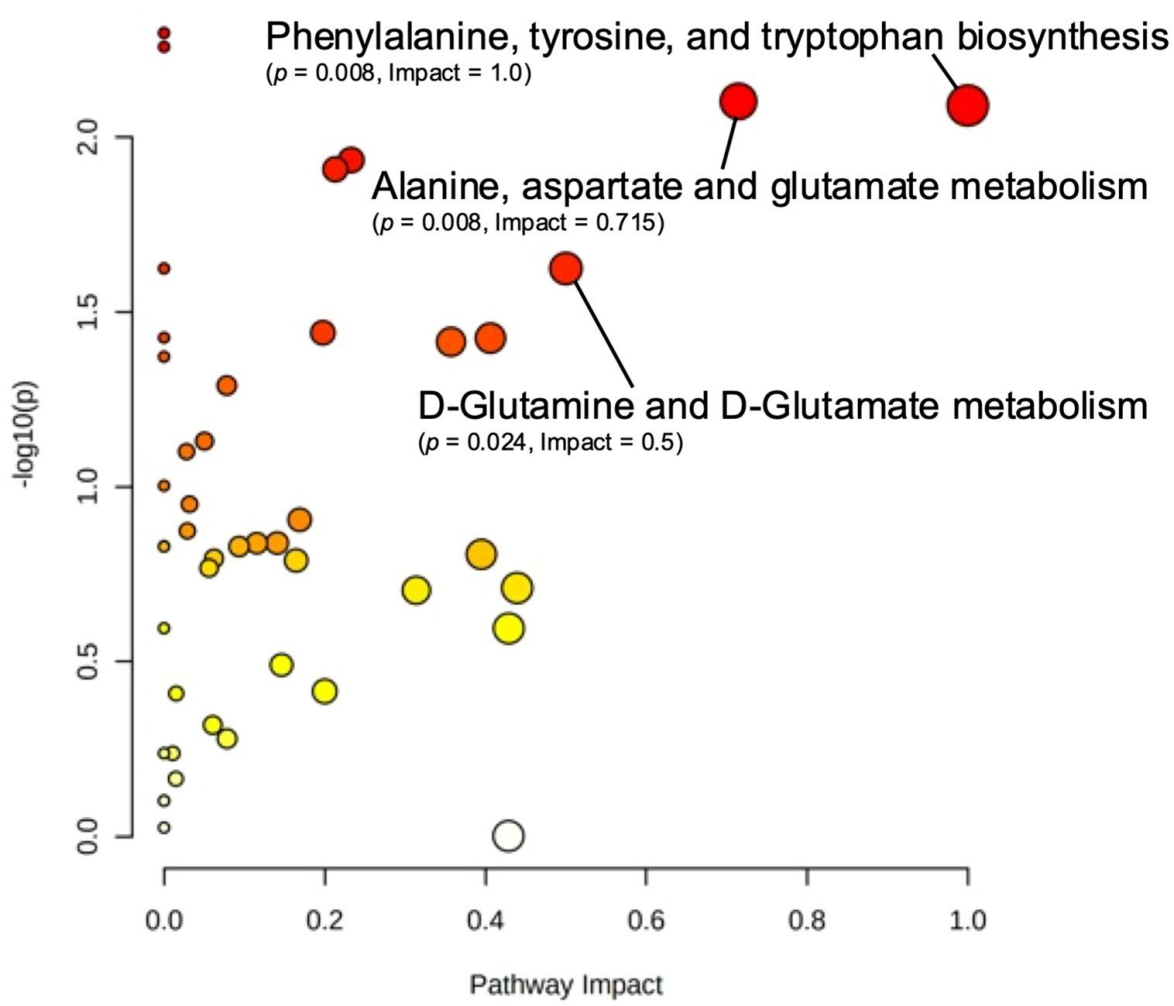
Scatter plot of pathway topology analysis. Metabolomic data were mapped to the KEGG human pathway library, and significance and impact were calculated using a global test of relative-betweenness centrality. Pathways with high impact (≥ 0.50) and significance (*p* < 0.05) have been labeled.

Pathway topology analysis was conducted to predict pathway enrichment between study groups. Metabolomic data were mapped to the KEGG human pathway library, and significance and impact were calculated using a global test of relative-betweenness centrality. A scatter plot view of the results can be seen in Fig. 8. Three pathways were observed to have both high impact (≥ 0.50) and significance (*p* < 0.05): phenylalanine (pathway impact = 1.0, *p* = 0.008), alanine, aspartate, and glutamate metabolism (pathway impact = 0.715, *p* = 0.008), and D-glutamine/D-glutamate metabolism (pathway impact = 0.50, *p* = 0.024). All reliably detected metabolites in these pathways, with the exception of GABA, were decreased in the high screen time group.

### Integrative analysis of multi-omics data revealed disturbances in amino acid metabolism

We used mmvec^36^ to integrate 16S sequencing and metabolomic data to assess cooccurrence patterns between GM features (genera) and fecal metabolites. We found that *Ruminococcaceae*, previously identified by Songbird analysis as the taxa most strongly associated with low screen time, had a high probability of co-occurring with five amino acids: isoleucine, l-alloisoleucine/leucine/norleucine, valine, proline, and phenylalanine. Upon visual examination of the biplot, we noted taxa with the highest cooccurrences were *Collinsella*, *Lactobacillales*, *Ruminococcus*, *cc115* (family *Erysipelotrichaceae*), and *Turicibacter* (Supplementary Fig. S7). These taxa were also highly co-occurrent with the same five amino acids (Supplementary Table S5).

### Predicted functional results were synthesized to form an integrated hypothesis describing the potential pathophysiology underlying high screen time

**Figure 9:**
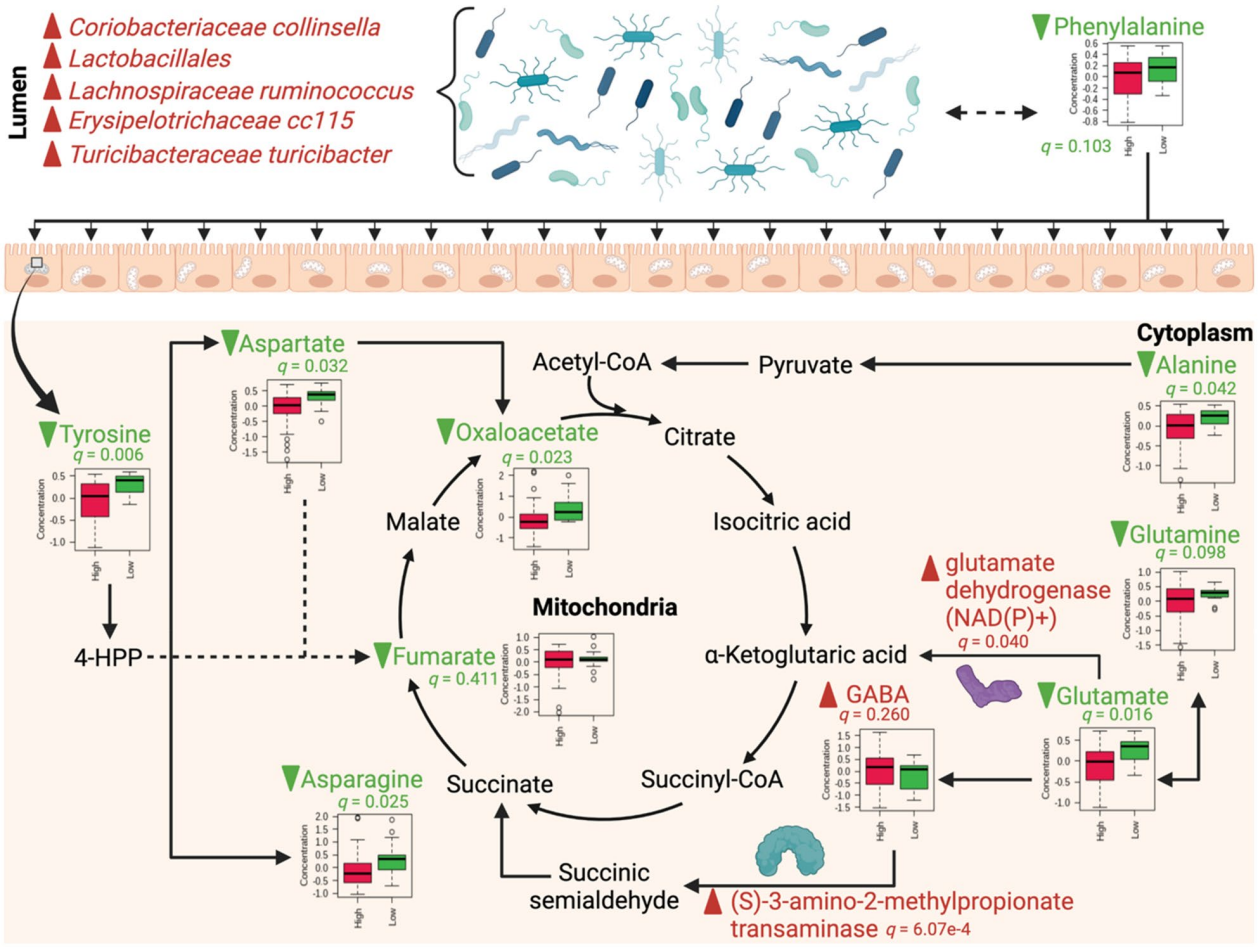
Integrated hypothesis of the potential pathophysiology underlying high screen time. The conceptual schema was synthesized using results of pathway topology analysis, PICRUSt analysis, and mmvec analysis. Green lettering with downward green arrows denotes decreases in the high screen time group, while red lettering and upward red arrows denotes increases in the high screen time group; box plots and significance information is provided where appropriate. Significance of plotted metabolites derived from independent *t*-test with FDR correction. Created with BioRender.com.

Our findings are conceptualized with respect to central carbon metabolism in Fig. 9. Three canonical KEGG pathways were indicated by pathway topology analysis as significantly impacted (0.008 ≤ *p* ≤ 0.024): (1) phenylalanine, tyrosine, and tryptophan biosynthesis, (2) alanine, aspartate, and glutamate metabolism, and (3) d-glutamine and d-glutamate metabolism. Between these pathways, 10 metabolites were detected in the current study; of these, six were significantly different between groups as determined by *t*-test with FDR correction (0.006 ≤ *q* ≤ 0.042). Nine of these pathway-embedded metabolites were reduced in the high screen time group (alanine, asparagine, aspartate, fumarate, glutamate, glutamine, oxaloacetate, phenylalanine, tyrosine), while one metabolite was elevated with high screen time (GABA). Importantly, PICRUSt analysis predicted the mean proportions of two enzymes in these pathways to be significantly greater with high screen time. Namely, those were (S)-3-amino-2-methylpropionate transaminase (*q* = 6.07e−4) and glutamate dehydrogenase (*q* = 0.040). Additionally, when results of our mmvec analysis were incorporated into our hypothesis, a high co-occurrence of five bacterial species with one pathway-embedded metabolite (phenylalanine) was revealed. This indicates a high probability of microbe-metabolite interaction between phenylalanine and the subset of microbial consortia (*C. collinsella*, *E. cc115*, *L. ruminococcus*, *Lactobacillales*, and *T. turicibacter*). Higher abundance of all five bacteria was observed in the high screen time group. Box plots and significance information are included in Fig. 9 where appropriate.

## Discussion

Screens are highly integrated in every facet of modern life, especially for young people^1,2^. Yet, despite growing concern over the detrimental effects of high screen time, previous studies have largely focused on the cognitive and behavioral effects of screen use and have exclusively relied on self-report data^8–11^, with only a few studies utilizing imaging techniques^14–17^. In this study, we explored the fecal microbiome and metabolome of a diverse group of college students, classified by high (≥ 75 min/day) or low (0–75 min/day) screen time. The taxonomic profile of the microbiome, but not overall diversity, modestly differed between the two classifications of screen time. Indeed, several health-associated microbes such as *Bacteroides*, *Akkermansia*, *Alistipes*, *Ruminococcus*, *Sutterella*, *Oscillospira*, and *Methanobrevibacter* were found to be more associated with low screen time, even after accounting for physical activity, sex, BMI, and age. This finding suggests that compositional differences may occur based on daily screen time. Meanwhile, the metabolome proved to be a sensitive marker of screen time; a panel of five metabolites were significant lower in the high screen time group. Predicted functional analysis of the microbiome revealed significant enrichment of numerous enzymes and taxa associated with various disease, diet, and lifestyle factors. Predicted metabolomic states indicated enrichment of several other enzymes, pathways, and disease profiles. Cumulatively, our results revealed dysfunction of amino acid metabolism which related to several key taxa.

By comparing the log ratio abundances of screen-time associated taxa, we noted *Ruminococcaceae* was one of the features most associated with low screen time. This family of bacteria has been positively correlated with lower risk of weight gain and improved energy metabolism in mice^37^ and a reduction of metabolic syndrome in humans^38^. Against the top associated taxa with high screen time, we noted significance between log ratio abundances in some important instances. Specifically, a greater ratio of *Peptostreptococcaceae/Runimococcaceae* in those with a high screen time classification was observed. *Peptostreptococcaceae* has been found to be abundant in patients with bile duct and colorectal cancers^39,40^. In addition, the low screen time classification had a significantly greater *Akkermansia*/*Bacteroides* ratio. *Akkermansia* has been correlated to several health states^41^, such as being positively associated with an increase in insulin sensitivity^42^. More recently, the species *Akkermansia muciniphila* has been explored for use as an oral probiotic therapy for obesity and metabolic syndrome^43^. A genus that was found to be more associated with high screen time was *Prevotella*. This finding was intriguing, given that *Prevotella* has been considered beneficial due to its abundance in the GM of healthy individuals and associations with plant-rich diets and weight loss^44,45^. Moreover, *Prevotella* has been reported to be more common in non-Westernized populations^46,47^. However, *Prevotella* is a large genus with high species diversity and high genetic diversity between strains^48^. For example, several *Prevotella* species have been suggested as intestinal pathobionts^49^. *Prevotella* has also been found to be increased in individuals with metabolic syndrome^50^. Another ratio analyzed, *Prevotella*/*Bacteroides*, which have been promoted as potential biomarkers for diet and lifestyle^51^, did not show significance between classifications, suggesting that more evidence is needed to establish a predictive relationship.

We performed a prediction of function analysis using microbiome data, identifying significant differences in pathway enrichment between low and high screen time classification. The top differential predicted functions were significantly more enriched in the high screen time classification. These included enzymes involved in energy metabolism, such as CoA-transferases, carboxylases, and dehydrogenases. The most significant predicted function, glutaconate CoA-transferase, is responsible for producing acetate and glutaconyl-1-CoA^52^. Acetate may originate from microbial fermentation of residual peptides and fats^53,54^. In a Western diet with low fiber intake, protein fermentation occurs mainly in the distal colon when saccharolytic substrates have been depleted^55^. Participants in both classifications had fiber consumptions well below the AMDR. Increased protein fermentation can produce amino acids which can further be metabolized via cross-feeding mechanisms and alter gut integrity and insulin sensitivity^56^. Upon performing microbe-metabolite cooccurrence analysis we noted multiple amino acids with the great probability to occur with the taxa *Collinsella*, *Lactobacillales*, *Ruminococcus*, *cc115*, and *Turicibacter*. Although these did not differ significantly by screen time classification, they were all elevated in the high screen time group.

The metabolome also proved to differ by screen time. An age, sex, and BMI-controlled GLM showed significant disturbances (*q* < 0.05) in five metabolites: 1-methylhistidine, alanine, proline, picolinic acid, and tyrosine. Although no previous studies have shown any associations between screen time and levels of 1-methylhistidine, proline, picolinic acid or tyrosine, alanine has been previously linked to high screen time and increased metabolic risk factors^57,58^. Among adolescents, reduced alanine via increased alanine transaminase (ALT) activity was associated with high screen time, higher blood glucose, and reduced insulin sensitivity^57^. A similar association was observed between ALT and sedentary behavior in a cohort of obese 11–13-year-olds^58^. In the current study, levels of alanine and glutamate (both substrates and products of the reversible ALT reaction) were significantly lower in the high screen time group (0.016 ≤ *q* ≤ 0.042). Future studies investigating objective markers of screen time for targeted therapy or surveillance monitoring should target the metabolome given its superior sensitivity compared to the more idiosyncratic changes of the microbiome.

Metabolome data was also mapped to canonical KEGG pathways to perform enzyme and pathway enrichment analyses. Additionally, database searches of fecal metabolites were conducted in order to construct predicted disease profiles with regard to screen time status. Three pathways were significantly enriched in response to screen time: (1) phenylalanine, tyrosine and tryptophan biosynthesis (*p* = 0.008), (2) alanine, aspartate and glutamate metabolism (*p* = 0.008), and (3) glutamate and glutamine metabolism (*p* = 0.024). This is the first reported association between screen time and differential enrichment of these pathways. Enzyme enrichment analysis returned 22 enzymes predicted to be significantly dysfunctional between groups, two of which were corroborated by PICRUSt analysis of microbiome data: glutamate dehydrogenase (*q* = 0.040) and (S)-3-amino-2-methylpropionate transaminase (*q* = 6.07e−4). The current study is the first to report predicted functional changes in these enzymes in response to high screen time. Analysis of predicted disease profiles showed significant enrichment of metabolites related to various gastrointestinal disorders such as celiac disease, inflammatory bowel disease, treated celiac disease, and various forms of diverticular disease (0.001 ≤ *p* ≤ 0.031). Metabolite profiling of disease states also showed significant enrichment of metabolite sets related to obesity (*p* = 0.016) and chronic fatigue syndrome (*p* = 0.043). Many previous studies have demonstrated a strong, positive association between screen time and obesity risk and incidence^4,5,10,11,17,57^, while others have shown similarly strong associations between screen time, disordered sleep and fatigue^4,5,8–10,17,57^. Although no previous studies have indicated potential effects of screen time on GI diseases, many metabolites flagged as significant in the current study have been previously implicated in the onset and progression of GI disorders. For instance, levels of alanine and severity of celiac hepatitis are inversely correlated^59^, and proline catabolism has been shown to cause hypoxia and bowel inflammation^60^. Our results warrant further investigation into metabolite-driven changes in pathways relevant to GI disorders, with special regard to inflammatory conditions.

Cumulatively, we performed six discrete analyses of predicted functional changes in pathways and enzymes, as well as disease, diet, and lifestyle profiles. Notably, we also performed integrative analysis of microbiomic and metabolomic data using mmvec to identify cooccurrence patterns. Results of our pathway and enzyme enrichment analyses, PICRUSt analysis, and mmvec analysis are highly commensurate with one another and have been synthesized as an integrative hypothesis of predicted functional changes in Fig. 9. Interestingly, many changes predicted by our hypothesis have been previously implicated in disease, diet and lifestyle conditions noted by our TSEA and disease analysis of fecal metabolites. For instance, increased levels of *Lactobacillales* have been found in the stool of patients with chronic fatigue syndrome (CFS)^61^. In the current study, disease enrichment analysis predicted CFS to be significantly increased in the high screen time group (Fig. 7) and levels of *Lactobacillales* were also elevated in the high screen time group. Similarly, inverse associations between circulating levels of glutamate and tyrosine with clinical severity of CFS has previously been shown^62^; in our study, levels of both metabolites were significantly reduced in the high screen time group. Additionally, over-representation of *Lachnospiraceae* has been cited as a hallmark of inflammatory bowel conditions^63,64^. In our study, TSEA indicated significant enrichment of taxa related to Crohn’s disease in the high screen time group (Supplementary Fig. S4) and disease enrichment of fecal metabolites revealed significant enrichment of metabolite sets linked to celiac disease, inflammatory bowel disease, and various forms of diverticular disease (Fig. 7).

While this explorative study provides the foundation for more directed research, it has some limitations, particularly that we had a relatively small number of participants (*n* = 60), who were categorized according to self-reported screen-time. Furthermore, 46 subjects were classified into high screen time, while 14 subjects were classified into low screen time. Future studies should make efforts to enroll more low screen time participants. Although the present study uses cross-sectional data with concurrent microbiomic and metabolomics data to explore actual and predicted effects of high screen time, future studies should validate these findings using longitudinal repeated-measures designs. Also, this study is limited to young, healthy college students living in the United States. Expanding this sample to a wider range of populations would allow us to capture a greater diversity of screen time, physical activity, and dietary data, and to better assess potential associations in microbial communities and metabolic signatures. Additionally, we did not produce strain-level resolution based on our sequencing methodology, though assessment at the species level in the future is warranted, as is metagenomic function.

Nevertheless, this is the first study to investigate the effects of screen time at the molecular level. We report the association of screen time with fecal microbiome and metabolome profiles in a cohort of healthy young college students. Significant features of these profiles may prove to be powerful therapeutic targets for the deleterious effects of increased screen time. Importantly, high screen time was found to be associated with reduced abundance of commensal bacteria *Ruminococcaceae* and *Akkermansia*. Moreover, the health-promoting taxa *Bifidobacterium* and *Faecalibacterium* were most predictive of low screen time. High screen time was predicted to have an increase in energy metabolism pathways, which may be suggestive of increased energy harvesting in the gut. Likewise, numerous metabolites associated with health in humans were significantly decreased in the high screen time group, suggesting an important role of the metabolome in the propagation of screen time-associated pathogenesis. Importantly, we present an integrated hypothesis of widespread amino acid dysfunction and specific microbial-metabolite interactions using KEGG pathways, database searches, and advanced machine learning methods which deserves increased research given its ubiquitous indication in the current study.

## Methods

### Participants and study procedure

This study included data from 60 college students who were recruited from select on-campus dormitories at Arizona State University (ASU). Specifically, samples were obtained from a larger cross-sectional study that utilized mobile ecological momentary assessment methodology to assess the influence that social networks have on physical activity, dietary intake, and body weight in two residence halls at ASU in Tempe, Arizona^65^. Briefly, healthy college students living in on-campus housing, who were English speaking, and at least 18 years of age were eligible to participate. Exclusion criteria for this study included a history of malabsorptive disorders, high blood pressure, eating disorders, HIV infection, diabetes, and/or the use of antibiotics, antifungals, or probiotics in the 2 to 3 months prior to the study. This study was conducted during the Fall 2014 and Spring 2015 semesters. The ASU Institutional Review Board approved this study (STUDY00002019), and all participants provided written informed consent. All research protocols were conducted in accordance with the principles expressed in the Declaration of Helsinki.

Enrolled participants were provided with questionnaires capturing demographic, physical activity, and dietary intake data^27,65^. Height and weight were measured by trained research staff and BMI was calculated and participants were categorized based on the CDC guidelines^66^. Physical activity was assessed via the Godin-Shephard Leisure-Time Physical Activity Questionnaire^67^. Daily physical activity was calculated and MVPA was computed by summing the total time spent on strenuous and moderate activity. This validated survey for measuring physical activity habits in college-aged males and females also captured sedentary activity including screen time (excluding time in class and being physically active). Participants were asked to report average daily screen time categorically (i.e., 0–15 min, 15–75 min, 75–195 min, 195–315 min, 315–360 min, or 360 + min). Given previous screen time recommendations in children and adolescents by the WHO^12^ and the AAP^13^, as well deleterious effects associated with 120 min of screen time in adults^2^, we split participants at 75 min of self-reported screen time, as it conforms best to current knowledge and guidance. The ASA24 24 h dietary recall was used to assess students’ habitual dietary intake. Participants were also asked to complete three days of dietary recall (two weekdays and one weekend day). Days of intake were dropped if caloric intake was below 500 or in excess of 5000 kcal. If a participant did not have at least one day of adequate dietary intake they were excluded from the study. Using data from the ASA24–2014 Daily Total Nutrients Analysis File (TN), we examined total grams of protein, fat, carbohydrates, and fiber.

### Sample collection

The sample collection procedure used in this study has been previously reported^27^. Each study participant was provided with a fecal sample collection kit (Commode Specimen Collection Kit, Fisher Scientific, Anthem, AZ) in order to provide a single fecal sample for analysis. Collection kits were distributed to participants in small insulated cooler bags containing ice packs to keep samples cold while in transit post-collection. Before participants left with the kit, a brief demonstration on how to collect the sample was provided along with a sheet of instructions inside the cooler bag. Participants were asked to freeze their ice packs immediately so that they were frozen at the time of sample collection. Ice packs were rated to stay frozen for 36–48 h in an insulated container. All stool samples were retrieved from participants and delivered to the clinical research facility within 24 h of collection. Stool samples were stored at − 80 °C to preserve the microbial community.

### Microbiome analysis

#### DNA isolation, preparation, and sequencing

Assessment of the intestinal microbiome from fecal collections was carried out at the Biodesign Institute at ASU in Tempe, Arizona. Extraction of microbial DNA from fecal samples was accomplished using the PowerSoil DNA isolation kit as described by the manufacturer (MoBio Laboratories Ltd., Carlsbad, CA) using a beadbeater (BioSpec, Bartlesville, OK). Amplification and sequencing of variants was performed as previously described^27^.

#### Sequence data and statistical analysis

Raw 16S rRNA sequencing data for all samples have been deposited in the open-source repository “NCBI/Sequence Read Archive (SRA)” under project PRJNA473006 with accession numbers: SAMN09258197 – SAMN09258278 (https://www.ncbi.nlm.nih.gov/sra). In the present study, paired-end, demultiplexed data were imported and analyzed using QIIME 2 software version 2021.2^68^. Briefly, sequencing generated 5,259,656 reads with a median of 80,443 reads per sample. After viewing sequence quality plots based on 10,000 randomly selected reads, the first 13 bases of the forward and reverse reads were trimmed. Next, QC was performed via the DADA2 denoise-paired method to remove low quality regions and construct a feature table using amplicon sequence variants (ASVs). The feature-classifier plugin was used to classify ASVs taxonomically. A naive Bayes machine-learning classifier was pre-trained to differentiate taxa present in the 99% Greengenes 13_8 reference set trimmed to 250 bp of the V4 hypervariable region (corresponding to the 515F-806R primers) was used. This classifier works by identifying *k*-mers that are diagnostic for particular taxonomic groups and using that information to predict the taxonomic affiliation of each ASV^69^. Before constructing the taxonomic bar chart, we first filtered out any samples with fewer features than our rarefaction threshold (10,000) and then filtered out low abundance/low prevalence ASVs using the feature-table plugin with the filter-samples method. A phylogenic tree was then constructed using the fragment-insertion plugin where the sequences were inserted into the Greengenes 13_8 99% identity reference tree backbone. To account for uneven sequencing depth between samples, normalization was performed via alpha rarefaction. Based on the ASV feature table, a p-min-depth of 10 and a p-max-depth of 70,000 was used.

Diversity analysis was conducted with the diversity plugin. Αlpha diversity (intra-community diversity) was measured using richness (Shannon, Faith’s PD and observed ASVs) and evenness (Pielou) indexes and compared with Kruskal–Wallis tests. Beta diversity (inter-community diversity) was measured using Jaccard, Bray–Curtis, Unweighted UniFrac distance (qualitative measure), and Weighted UniFrac distance (quantitative measure). Adonis analyses with the covariates age, sex, MVPA, and BMI, were used to test for significant differences between high and low screen time for beta diversity metrics.

Relative abundance of taxa for high and low screen time classification were calculated at the genus level. The distinctive taxa between screen time classification were identified first through DEICODE, a compositionally aware form of Aitchison distance that is robust to high levels of sparsity^30^. This was conducted with the DEICODE plugin for QIIME 2 and the output was visualized as a robust Aitchison PCA, based on the feature table. To test significance using a multivariate model, the Adonis test in QIIME 2 was used to assess significant differences between screen time classification with the covariates sex, age, BMI, and MVPA time. Next, Songbird v1.0.1^31^ in QIIME 2 version 2020.6 was used to identify feature ranks (parameters: − p-epochs 10,000 − p-differential-prior 0.5 − p-summary-interval 1 − num-random-test-examples 10% of samples) based on screen time and accounting for the covariates sex, BMI, age, and MVPA time. Qurro v0.4.0^32^ was then used to compute log ratios of ranked features. Evaluation of the Songbird models against a baseline model obtained a pseudo-Q^2^ value of − 0.102, suggestive of possible overfitting. The top 20 highest and lowest ranked differential features were selected based on screen time classification. *T*-tests (Mann–Whitney *U* tests) and Cohen’s *d* were calculated to assess the significance (alpha = 0.05) and effect size of the log ratios.

In order to predict the function of fecal microbiota, data analysis was performed through the PICRUSt 2 pipeline^33^. Then, PICRUSt output for the level 3 KEGG entries were analyzed and illustrated with statistical analysis of the taxonomic and functional profiles (STAMP) software version 2.1.3^70^. Welch’s *t*-test was used to test for significant features between screen time classification with *p*-value corrections performed using Storey's FDR approach.

Taxon set enrichment analysis (TSEA) of disease, diet and lifestyle, in addition to core statistical and visual analysis of microbiome data, was performed using MicrobiomeAnalyst v1.0^35^.

### Metabolomics analysis

#### Reagents

Acetonitrile (ACN), methanol (MeOH), and ammonium acetate (NH_4_Oac), all LC–MS grade, were purchased from Fisher Scientific (Pittsburgh, PA). Ammonium hydroxide (NH_4_OH), O-methylhydroxylamine hydrochloride (MeOX), and N-Methyl-n-(tert-butyldimethylsilyl) trifluoroacetamide (MTBSTFA) were bought from Sigma-Aldrich (Saint Louis, MO). Deionized water was provided in-house by a water purification system from EMD Millipore (Billerica, MA). Phosphate buffered saline (PBS) was bought from GE Healthcare Life Sciences (Logan, UT). Standard compounds corresponding to measured aqueous metabolites/features were purchased from Sigma-Aldrich and Fisher Scientific. Lipid standards were purchased from Fisher Scientific, Sigma-Aldrich, and Avanti Polar Lipids (Alabaster, AL).

#### Targeted LC–MS/MS aqueous profiling

Prior to LC–MS/MS targeted measurement, frozen fecal samples were first thawed overnight under 4 °C. Afterward, 20 mg of each sample were placed in a 2 mL Eppendorf vial. The initial step for protein precipitation and metabolite extraction was performed by adding 500 μL MeOH and 50 μL internal standard solution (containing 1,810.5 μM ^13^C_3_-lactate and 142 μM ^13^C_5_-glutamic acid). The mixture was then vortexed for 10 s and stored at − 20 °C for 30 min, followed by centrifugation at 14,000 RPM (21,913×*g*) for 10 min at 4 °C. The supernatants (450 μL) were collected into new Eppendorf vials and dried using a CentriVap Concentrator (Fort Scott, KS). The dried samples were reconstituted in 150 μL of 40% PBS/60% ACN and centrifuged again at 14,000 RPM (21,913×*g*) at 4 °C for 10 min. Afterward, 100 μL of supernatant was collected from each sample into an LC autosampler vial for subsequent analysis. A pooled sample, which was a mixture of all experimental samples, was used as the QC sample and injected once every 10 experimental samples.

The targeted LC–MS/MS method used here was modeled after that developed and used in a growing number of studies^71–73^. Briefly, all LC–MS/MS experiments were performed on an Agilent 1290 UPLC-6490 QQQ-MS system. Each supernatant sample was injected twice, 10 µL for analysis using negative ionization mode and 4 µL for analysis using positive ionization mode. Both chromatographic separations were performed in hydrophilic interaction chromatography mode on a Waters Xbridge BEH Amide column (150 × 2.1 mm, 2.5 µm particle size; Waters Corporation, Milford, MA). The flow rate was 0.3 mL/min, auto-sampler temperature was kept at 4 °C, and the column compartment was set to 40 °C. The mobile phase was composed of Solvents A (10 mM NH_4_Oac, 10 mM NH_4_OH in 95% H_2_O/5% ACN) and B (10 mM NH_4_Oac, 10 mM NH4OH in 95% ACN/5% H_2_O). After an initial 1 min isocratic elution of 90% B, the percentage of Solvent B decreased to 40% at t = 11 min. The composition of Solvent B was maintained at 40% for 4 min (t = 15 min), after which the percentage of B gradually went back to 90%, to prepare for the next injection. The mass spectrometer was equipped with an electrospray ionization (ESI) source. Targeted data acquisition was performed in multiple-reaction-monitoring (MRM) mode. For targeted data acquisition, we monitored 118 and 160 MRM transitions in negative and positive mode, respectively (278 transitions in total). The whole LC–MS system was controlled by Agilent MassHunter Workstation software. The extracted MRM peaks were integrated using Agilent MassHunter Quantitative Data Analysis software.

#### GC–MS analysis of short chain fatty acids (SCFAs)

Frozen fecal samples were first thawed overnight under 4 °C. Afterward, 20 mg of each sample was homogenized with 5 μL hexanoic acid-3,3,3 (internal standard), 15 μL sodium hydroxide (NaOH [0.5 M]), and 500 μL MeOH. Following storage at − 20 °C for 20 min and centrifugation at 14,000 RPM (21,913×*g*) for 10 min, 450 μL of supernatant were collected and sample pH was adjusted to 10 by adding 30 μL of NaOH:H_2_O (1:4, v:v). Samples were then dried, and the residues were first derivatized with 40 µL of 20 mg/mL MeOX solution in pyridine under 60 °C for 90 min. Next, 60 µL of MTBSTFA containing d_27_-mysristic acid were added, and the mixture was incubated at 60 °C for 30 min. The samples were then vortexed for 30 s, followed by centrifugation at 14,000 RPM (21,913×*g*) for 10 min. Finally, 70 µL of supernatant were collected from each sample into new glass vials for GC–MS analysis.

GC–MS conditions used here were adopted from a previously published protocol^74^. Briefly, GC–MS experiments were performed on an Agilent 7820A GC-5977B MSD system (Santa Clara, CA) by injecting 1 µL of prepared samples. Helium was used as the carrier gas with a constant flow rate of 1.2 mL/min. The separation of metabolites was achieved using an Agilent HP-5 ms capillary column (30 m × 250 µm × 0.25 µm). The column temperature was maintained at 60 °C for 1 min, increased at a rate of 10 °C/min to 325 °C, and then held at this temperature for 10 min. Mass spectral signals were recorded at an *m/z* range of 50–600. Data extraction was performed using Agilent MassHunter Profinder software. A batch recursive feature extraction algorithm for small molecules was used, and peaks were filtered so that only peaks with absolute height ≥ 1,000 counts were included. An RT tolerance of 0.10 min was established, and extraction was limited to the largest 1,000 compound groups. Results were filtered if the overall identification score was less than 75.

#### Metabolite data analysis

Following peak integration, metabolites were filtered for reliability and only those with QC CV < 20% and relative abundance of 1000 in > 80% of samples were retained for analysis. The data were log_10_-transformed and Pareto scaled prior to analysis. Linear modelling was performed using SPSS 28.0 (SPSS Inc., Chicago, IL). Multivariate statistical analyses were performed using open-source R software. Pathway and enzyme enrichment analysis of metabolomic data were performed and visualized using MetaboAnalyst v5.0^75^.

#### Multi-omics data analysis

In order to identify microbial features associated with screen time classification and the metabolites they might be producing, we measured probabilities of cooccurrence between observed species (based on metagenomic data) and all metabolites (as informed by the metabolomic analysis). For this analysis, we used mmvec v1.0.2, a neural network solution inspired from natural language processing, to build a log-transformed conditional probability matrix from each cross-omics feature pair and apply singular value decomposition in order to represent cooccurrence in the form of biplots^76^.

## Supplementary Information

Supplementary Information.

## Data Availability

Raw 16S rRNA sequencing data for all samples have been deposited in the open-source repository “NCBI/Sequence Read Archive (SRA)” under project PRJNA473006 with accession numbers: SAMN09258197–SAMN09258278 (https://www.ncbi.nlm.nih.gov/sra). All mass spectrometry data and deidentified subject metadata analyzed in this study have been deposited to Mendeley Data and are publicly available (https://doi.org/10.17632/nd64f8zchj.1).
