# Supplementary material for: Microbiome and metabolome profiles of high screen time in a cohort of healthy college students
Source: Sci Rep. 2022 Mar 2;12:3452. doi: 10.1038/s41598-022-07381-3 (PMC8891328; doi:10.1038/s41598-022-07381-3)
Supplement: Supplementary file 1 — Supplementary Information. [file 41598_2022_7381_MOESM1_ESM.pdf]

Supplementary Information

Microbiome and Metabolome Profiles of High Screen Time in a Cohort of Healthy College Students

Paniz Jasbi[1+], Alex E. Mohr[1+], Xiaojian Shi[1,2], Tara Mahmood[1,3], Qiyun Zhu[4,5], Meg Bruening[1], Haiwei Gu[1,6*], Corrie Whisner[1*]

[1]College of Health Solutions, Arizona State University, Phoenix, AZ, USA

[2]Systems Biology Institute, Yale University, West Haven, CT, USA

[3]Department of Nutrition, Texas A&M University, College Station, TX, USA

[4]School of Life Sciences, Arizona State University, Tempe, AZ, USA

[5]Biodesign Center for Fundamental and Applied Microbiomics, Arizona State University, Tempe AZ, USA

[6]Center for Translational Science, Florida International University, Port St. Lucie, FL, USA

[+]These authors contributed equally

*Corresponding authors

Email: hgu@fiu.edu; cwhisner@asu.edu

Page S-3, Table S1 Alpha and beta diversity metrics for low and high screen time classifications.

Page S-4, Table S2 Top 20 highest (set 1) and lowest (set 2) ranked ASVs associated with screen time classification as produced by Songbird analysis.

Page S-6, Table S3 Metabolite results of covariate controlled GLM with FDR adjustment for multiple comparisons.

Page S-11, Table S4 Significantly enriched enzymes as predicted by 912 metabolite sets.

Page S-12, Table S5 Taxa with greatest metabolite cooccurrence as produced by mmvec analysis.

Page S-13, Fig. S1 Two dimensional scores plot of PCA conducted using the entire set of captured metabolites and GM features between groups. Analysis shows two potential outliers (DW40 and DW100) that were removed prior to additional analyses.

Page S-14, Fig. S2 Heatmap of core microbiome. Results are displayed as prevalence by detection threshold at the genus level.

Page S-15, Fig. S3 Predicted functional metabolic pathways by high vs low screen time. **a** PCA analysis. **b** Scatter plot displaying the predicted functional differences between high vs low screen time. Gray lines indicate 95% CIs for each feature.

Page S-16, Fig. S4 TSEA of microbiome data. **a** Network view of 239 taxon sets associated with host-intrinsic factors such as disease. Significant taxon sets (*q*): liver cirrhosis (0.001), Crohn's disease (0.001), type I diabetes (0.003) overweight/obese mother (0.003), autism (0.006), myocardial infarction (0.008). Liver cirrhosis and autism were predicted to be decreased in high screen time group; Crohn's disease, type I diabetes, overweight/obese mother, and myocardial infarction were predicted to be increased in high screen time group. **b** Network view of 118 taxon sets associated with host-extrinsic factors including diet and lifestyle. Significant taxon sets (*q*): red wine (0.007), coffee (0.025), high fat diet (0.047). Consumption of a high-fat diet was predicted to be decreased in high screen time group; consumption of red wine and coffee were predicted to be increased in high screen time group.

Page S-17, Fig. S5 Distribution of CV values of all measured metabolites in this study. QC CV range: 1.25%-20.0%, median CV: 11.5%, with ~76% of metabolites showing CV < 15%.

Page S-18, Fig. S6 Enzyme enrichment analysis of metabolomic data performed using 912 metabolic sets predicted to change in the case of dysfunctional enzymes.

Page S-19, Fig. S7 Integrative analysis of 16S sequencing and metabolomic data sets. Taxa are represented by arrows indicating their co-occurrences with metabolites (dots).

Table S1 Alpha and beta diversity metrics for low and high screen time classifications.

Alpha diversity metric*	Low (*n* = 14)	High (*n* = 46)	*F*-statistic	*p*
Observed Features	100.07 ± 6.56	97.80 ± 4.14	0.104	0.748
Faith's PD	11.68 ± 0.77	11.25 ± 0.47	0.383	0.539
Pielou's E	0.74 ± 0.02	0.73 ± 0.01	0.173	0.679
Shannon	4.91 ± 0.16	4.86 ± 012	0.187	0.667

Beta diversity metric	Sum of Squares	R^2	*F*-statistic	*p*
Jaccard	0.271	0.015	0.867	0.855
Bray Curtis	0.345	0.022	1.175	0.219
Unweighted UniFrac	0.120	0.013	0.683	0.935
Weighted UniFrac	0.119	0.027	1.312	0.239

*Alpha diversity values for screen time classification displayed as mean ± SD.

Table S2 Top 20 highest (set 1) and lowest (set 2) ranked ASVs associated with screen time classification as produced by Songbird analysis.

Set 1	Feature	Intercept	Log FC (high/low)
1	k__Bacteria; p__Firmicutes; c__Clostridia; o__Clostridiales; f__Ruminococcaceae; g___	-2.116	3.103
2	k__Bacteria; p__Verrucomicrobia; c__Verrucomicrobiae; o__Verrucomicrobiales; f__Verrucomicrobiaceae; g__Akkermansia; s__muciniphila	-0.833	2.739
3	k__Bacteria; p__Bacteroidetes; c__Bacteroidia; o__Bacteroidales; f__Rikenellaceae; g__Alistipes; s__indistinctus	-0.393	2.735
4	k__Bacteria; p__Actinobacteria; c__Coriobacteriia; o__Coriobacteriales; f__Coriobacteriaceae; g__; s__	0.523	2.225
5	k__Bacteria; p__Firmicutes; c__Clostridia; o__Clostridiales; f__Christensenellaceae; g__; s__	1.82	2.078
6	k__Bacteria; p__Firmicutes; c__Clostridia; o__Clostridiales; f__Ruminococcaceae; g__; s__	1.599	1.900
7	k__Bacteria; p__Firmicutes; c__Clostridia; o__Clostridiales; f__Ruminococcaceae; g__Ruminococcus; s__	0.029	1.665
8	k__Bacteria; p__Firmicutes; c__Clostridia; o__Clostridiales; f__Ruminococcaceae	0.839	1.611
9	k__Bacteria; p__Proteobacteria; c__Betaproteobacteria; o__Burkholderiales; f__Alcaligenaceae; g__Sutterella; s__	-0.313	1.590
10	k__Bacteria; p__Firmicutes; c__Clostridia; o__Clostridiales; f__Ruminococcaceae; g__Ruminococcus; s__	0.541	1.587
11	k__Bacteria; p__Firmicutes; c__Clostridia; o__Clostridiales; f__; g__; s__	-0.872	1.569
12	k__Bacteria; p__Bacteroidetes; c__Bacteroidia; o__Bacteroidales; f__Rikenellaceae; g__; s__	-0.704	1.568
13	k__Bacteria; p__Firmicutes; c__Clostridia; o__Clostridiales; f__Ruminococcaceae; g__Oscillospira; s__	-0.549	1.533
14	k__Archaea; p__Euryarchaeota; c__Methanobacteria; o__Methanobacteriales; f__Methanobacteriaceae; g__Methanobrevibacter; s__	0.136	1.429
15	k__Bacteria; p__Firmicutes; c__Clostridia; o__Clostridiales; f__Ruminococcaceae	1.128	1.412
16	k__Bacteria; p__Bacteroidetes; c__Bacteroidia; o__Bacteroidales; f__Rikenellaceae; g__Alistipes; s__putredinis	0.751	1.366
17	k__Bacteria; p__Bacteroidetes; c__Bacteroidia; o__Bacteroidales; f__Bacteroidaceae; g__Bacteroides; s__	-0.187	1.338
18	k__Bacteria; p__Firmicutes; c__Clostridia; o__Clostridiales; f__Christensenellaceae; g__; s__	1.003	1.222
19	k__Bacteria; p__Firmicutes; c__Clostridia; o__Clostridiales; f__Christensenellaceae; g__; s__	-0.611	1.202
20	k__Bacteria; p__Firmicutes; c__Clostridia; o__Clostridiales; f__; g__; s__	1.516	1.169
**Set 2**			

1	k__Bacteria; p__Firmicutes; c__Clostridia; o__Clostridiales; f__Ruminococcaceae	-1.064	-1.389
2	k__Bacteria; p__Firmicutes; c__Clostridia; o__Clostridiales; f__Lachnospiraceae; g__Coprococcus; s__	-0.884	-1.402
3	k__Bacteria; p__Firmicutes; c__Clostridia; o__Clostridiales; f__Lachnospiraceae; g__Lachnospira; s__	0.823	-1.466
4	k__Bacteria; p__Firmicutes; c__Clostridia; o__Clostridiales; f__Ruminococcaceae; g__Oscillospira; s__	0.370	-1.473
5	k__Bacteria; p__Firmicutes; c__Clostridia; o__Clostridiales; f__Lachnospiraceae; g__[Ruminococcus]; s__gnavus	-1.452	-1.532
6	k__Bacteria; p__Bacteroidetes; c__Bacteroidia; o__Bacteroidales; f__Bacteroidaceae; g__Bacteroides	0.200	-1.564
7	k__Bacteria; p__Bacteroidetes; c__Bacteroidia; o__Bacteroidales; f__Bacteroidaceae; g__Bacteroides; s__ovatus	-1.580	-1.566
8	k__Bacteria; p__Firmicutes; c__Clostridia; o__Clostridiales; f__Lachnospiraceae; g__Coprococcus; s__	-1.356	-1.586
9	k__Bacteria; p__Firmicutes; c__Clostridia; o__Clostridiales; f__Lachnospiraceae	-0.950	-1.825
10	k__Bacteria; p__Firmicutes; c__Clostridia; o__Clostridiales; f__Lachnospiraceae; g__Coprococcus; s__eutactus	-1.224	-1.830
11	k__Bacteria; p__Firmicutes; c__Clostridia; o__Clostridiales; f__Lachnospiraceae; g__Coprococcus; s__	-1.343	-1.968
12	k__Bacteria; p__Bacteroidetes; c__Bacteroidia; o__Bacteroidales; f__Prevotellaceae; g__Prevotella; s__copri	-2.986	-2.021
13	k__Bacteria; p__Firmicutes; c__Clostridia; o__Clostridiales; f__Lachnospiraceae; g__Lachnospira; s__	-1.398	-2.105
14	k__Bacteria; p__Firmicutes; c__Clostridia; o__Clostridiales; f__Peptostreptococcaceae	-2.730	-2.144
15	k__Bacteria; p__Firmicutes; c__Clostridia; o__Clostridiales; f__Lachnospiraceae; g__Lachnospira; s__	-2.066	-2.194
16	k__Bacteria; p__Bacteroidetes; c__Bacteroidia; o__Bacteroidales; f__Bacteroidaceae; g__Bacteroides; s__fragilis	-1.511	-2.359
17	k__Bacteria; p__Bacteroidetes; c__Bacteroidia; o__Bacteroidales; f__Prevotellaceae; g__Prevotella; s__copri	-0.898	-2.391
18	k__Bacteria; p__Proteobacteria; c__Gammaproteobacteria; o__Pasteurellales; f__Pasteurellaceae	2.812	-2.905
19	k__Bacteria; p__Firmicutes; c__Clostridia; o__Clostridiales; f__Veillonellaceae; g__Veillonella; s__dispar	0.113	-3.506
20	k__Bacteria; p__Bacteroidetes; c__Bacteroidia; o__Bacteroidales; f__Prevotellaceae; g__Prevotella; s__	0.162	-5.22

Table S3 Metabolite results of covariate controlled* GLM with FDR adjustment for multiple comparisons.

Metabolite	Mean Difference (High-Low)	Std. Error	FDR *q*	95% Confidence Interval for Difference	
				Lower Bound	*Upper Bound*
1-Methylhistidine	-549695.4	162022.8	0.002	-877155.6	-222235.1
Alanine	-2129339.7	881912.0	0.020	-3911750.5	-346929.0
Proline	-14379814.1	6116346.8	0.024	-26741412.0	-2018216.2
Picolinic acid	-21815.4	9919.4	0.034	-41863.2	-1767.5
Tyrosine	-857502.8	420133.5	0.048	-1706624.2	-8381.4
Cytidine	52660.5	27792.9	0.065	-3510.9	108832.0
Sarcosine	-2073580.4	1098680.2	0.066	-4294095.8	146935.1
Xylose	-16900.1	9006.2	0.068	-35102.3	1302.1
TMAO	26289.4	14330.3	0.074	-2673.3	55252.1
p-Coumaric acid	-47262.4	26167.4	0.078	-100148.7	5623.9
Pregnenolone SO_4^{2-}	-95718.1	53455.9	0.081	-203756.5	12320.4
Hse/Thr	-904642.8	511225.8	0.084	-1937868.5	128583.0
Tryptamine	-329730.4	187888.4	0.087	-709467.0	50006.3
Glycine	-69662.2	40125.1	0.090	-150758.1	11433.8
L-Allo/(Nor)/Leucine	-9049177.6	5245200.2	0.092	-19650122.6	1551767.5
Glutamic acid	-2233732.8	1314685.6	0.097	-4890811.6	423345.9
Aspartate	-152271.6	90453.4	0.100	-335084.7	30541.6
2-Methylbutyric acid/Valeric acid	-5144603.0	3086140.8	0.103	-11381926.2	1092720.3
Myristic acid	-801562.8	488749.6	0.109	-1789362.6	186237.0
Nonadecanoic acid	1296505.1	796759.5	0.112	-313805.9	2906816.0
4-Aminobutyric acid	1127788.0	707365.4	0.119	-301850.8	2557426.8
Valeric acid	-0.1	0.1	0.131	-0.3	0.0
Pyridoxine	145086.0	94238.7	0.132	-45377.6	335549.6
Butyric acid	0.1	0.0	0.137	0.0	0.1
Isoleucine	-16958257.9	11218357.9	0.138	-39631405.0	5714889.1
2HG	-47154.4	31859.1	0.147	-111544.0	17235.3
Quinolinic acid	-7497.0	5086.8	0.148	-17777.7	2783.8
Tryptophan	-714253.1	488263.3	0.151	-1701070.0	272563.9
2-Aminoadipic acid	37176.0	26086.7	0.162	-15547.3	89899.2
2-Deoxyadenosine	36251.8	25485.4	0.163	-15256.1	87759.7
Phenylalanine	-3491118.1	2476801.7	0.166	-8496921.1	1514684.8

Adenosine	150139.9	109970.4	0.180	-72118.7	372398.4
Caprylic acid	140016.3	104682.5	0.189	-71554.9	351587.5
Hydroxyproline	-394411.2	295539.0	0.190	-991717.9	202895.5
2-Methylbutyric acid	-0.1	0.1	0.191	-0.2	0.0
5-Aminolevulinic acid	-421078.2	322583.3	0.199	-1073043.3	230886.9
Glycocyamine	162720.4	125032.3	0.201	-89979.4	415420.2
Valine	-4248474.6	3313587.3	0.207	-10945484.3	2448535.1
13C5-15N-Glutamic acid	84155.1	66799.1	0.215	-50850.9	219161.0
N-Acetylethanolamine	26653.5	21523.4	0.223	-16846.9	70153.9
Ornithine	-2242078.8	1822027.6	0.226	-5924533.9	1440376.4
Acetylglucosamine	-128259.4	106261.8	0.235	-343022.6	86503.8
Methionine	-931500.8	788127.8	0.244	-2524366.4	661364.8
2-deoxyguanosine	57411.8	49090.6	0.249	-41804.0	156627.5
Nicotinamide	3222.1	2895.4	0.272	-2629.8	9073.9
Acetic acid	0.6	0.6	0.279	-0.5	1.8
Glycylproline	-73625.9	69281.4	0.294	-213648.9	66397.0
Norvaline	-3704914.1	3495559.4	0.296	-10769703.2	3359875.0
Inosine	143769.3	135990.3	0.297	-131077.3	418615.9
Kynurenic acid	6236.4	5968.0	0.302	-5825.4	18298.3
Betaine	3478425.9	3350572.5	0.305	-3293333.7	10250185.5
Glutaconic acid	8861.6	8662.0	0.312	-8644.9	26368.2
2-Deoxycytidine	11492.3	11852.1	0.338	-12461.6	35446.3
3-Phenyllactic acid	-93646.5	97507.9	0.343	-290717.2	103424.3
Urocanic acid	-355547.3	370502.4	0.343	-1104360.5	393265.9
Pentadecanoic acid	-642643.4	697024.5	0.362	-2051382.5	766095.7
Indole-3-lactic acid	-20099.7	22008.0	0.367	-64579.6	24380.1
Mucic acid	33939.7	37705.0	0.373	-42265.0	110144.4
Heptadecanoic acid	134525.1	159849.9	0.405	-188543.6	457593.8
4-Imidazoleacetic acid	118024.4	142390.9	0.412	-169758.3	405807.2
Azelaic acid	103120.1	130507.2	0.434	-160644.8	366885.1
Indole-3-acetic acid	79977.4	101428.5	0.435	-125017.2	284972.0
Xanthine	-77719.5	109066.1	0.480	-298150.2	142711.2

Acetyl-L-glutamine	-15033.8	21251.2	0.483	-57984.1	27916.5
alpha-Ketoisovaleric acid/Maleic acid	7580.9	11046.3	0.496	-14744.5	29906.3
2/3-(OH)phenylacetic acid	13609.8	20158.7	0.503	-27132.5	54352.1
alpha-KG/Adipic acid	4671.4	6990.4	0.508	-9456.7	18799.4
2/3-Aminoisobutyric acid/Dimethylglycine	32676.3	48898.3	0.508	-66150.8	131503.3
Nicotinic acid	84216.3	127348.9	0.512	-173165.4	341597.9
Guanosine	22369.3	33911.6	0.513	-46168.6	90907.2
Phenylacetic acid	-30719.9	46670.8	0.514	-125045.0	63605.2
Trehalose	-6944.2	10919.8	0.528	-29013.9	15125.5
Methylguanidine	-27668.2	47013.8	0.559	-122686.6	67350.3
2-Methylglutaric acid	-18215.9	31106.3	0.561	-81084.1	44652.4
Uridine	-4872.7	8933.2	0.588	-22927.4	13181.9
3-Methyladipic acid	14691.1	27348.9	0.594	-40583.0	69965.2
Fumarate	5997.3	11305.4	0.599	-16851.8	28846.3
Adenine	87233.9	165459.2	0.601	-247171.5	421639.3
Cytosine	197315.2	374208.0	0.601	-558987.3	953617.7
4-Hydroxybenzaldehyde	-51246.7	97870.5	0.603	-249050.4	146556.9
Guanine	175637.4	339487.8	0.608	-510493.0	861767.7
3-hydroxykynurenine	530.7	1048.6	0.616	-1588.7	2650.1
Glutamine	-217753.8	434878.1	0.619	-1096675.2	661167.6
5-Hydroxytryptophan	-14583.6	29518.1	0.624	-74241.9	45074.7
dTMP	849.4	1755.1	0.631	-2697.7	4396.6
3-Aminobutyric acid	-24508.1	50992.8	0.633	-127568.5	78552.2
Citrulline	-223547.8	472104.0	0.638	-1177705.6	730609.9
6-Methyl-DL-Tryptophan	3385.6	7352.6	0.648	-11474.7	18245.9
9-Octadecynoic acid	-717273.2	1582598.6	0.653	-3915824.2	2481277.8

Amino valerate	363235.7	815813.4	0.659	-1285584.8	2012056.1
Lactate	168916.2	381537.8	0.660	-602200.5	940032.9
2-Deoxyuridine	-1274.9	2885.6	0.661	-7106.9	4557.1
Propionic acid	0.2	0.5	0.661	-0.8	1.3
Urate	1053.5	2405.4	0.664	-3807.9	5915.0
Asparagine	-25377.5	59985.6	0.675	-146612.8	95857.9
Isovalerylglycine	-1264.0	3035.0	0.679	-7398.0	4870.0
Citraconic acid	2650.3	6419.6	0.682	-10324.3	15624.8
Sebacic acid	16378.5	40270.3	0.686	-65010.7	97767.7
Acetylcarnitine	-259110.0	656469.5	0.695	-1585884.4	1067664.3
Serine	-65624.4	170775.7	0.703	-410775.0	279526.2
Pipecolinic acid	-511989.4	1391781.8	0.715	-3324885.4	2300906.6
2-Hydroxybenzoic acid	-2391.9	6657.9	0.721	-15847.9	11064.2
4-Hydroxyphenylacetic acid/Mandelic acid	6998.2	20225.6	0.731	-33879.2	47875.7
Capric acid	69384.0	203918.1	0.735	-342749.9	481517.9
Biotin	-11415.6	34883.2	0.745	-81917.3	59086.0
Aconitic acid	-1328.5	4063.7	0.745	-9541.5	6884.4
2-Aminobutyric acid	-515472.3	1595185.2	0.748	-3739461.8	2708517.3
Heptanoic acid	0.0	0.0	0.751	-0.1	0.1
Phosphocreatine	17702.1	56413.8	0.755	-96314.6	131718.7
Creatinine	-488413.2	1571477.6	0.758	-3664487.8	2687661.4
Acetylcholine	104056.5	353273.0	0.770	-609934.9	818047.8
Gentisic acid	1167.5	4136.0	0.779	-7191.6	9526.7
Acetylornithine	116741.1	428108.8	0.786	-748499.0	981981.2
5-Methylcytidine	187.9	691.3	0.787	-1209.2	1585.1
NAD	-1477.7	5834.9	0.801	-13270.5	10315.1
Lactose	-10394.0	42761.0	0.809	-96817.2	76029.3
Pyroglutamic acid	801005.6	3337864.1	0.812	-5945069.4	7547080.5
13C3-Lactate	11636.1	50153.4	0.818	-89727.7	112999.8
Carnosine	33630.9	151381.2	0.825	-272321.9	339583.7
4-Methyl-2-oxopentanoic	1309.7	6897.4	0.850	-12630.3	15249.8

acid/Ketoleucine/Ketoisoleucine					
Carnitine	270064.3	1446361.8	0.853	-2653141.9	3193270.5
3-Methyl-2-oxovaleric acid	43649.9	238712.6	0.856	-438806.4	526106.1
Levulinic acid	189.3	1112.4	0.866	-2058.9	2437.5
2,3-Dihydroxybenzoic acid	421.4	2663.0	0.875	-4960.8	5803.5
Lauric acid	178872.0	1156088.3	0.878	-2157669.7	2515413.6
Glutaric acid	26150.2	186488.4	0.889	-350756.9	403057.3
Protocatechuic acid	1627.5	12380.5	0.896	-23394.4	26649.3
2-Pyrrolidinone	-802.9	6874.0	0.908	-14695.9	13090.0
Stearic acid	180988.0	1867599.7	0.923	-3593571.8	3955547.9
Palmitic acid	-93821.4	1058111.2	0.930	-2232343.9	2044701.1
Caproic acid	0.0	0.1	0.953	-0.1	0.1
Xanthosine	-632.8	12959.3	0.961	-26824.6	25558.9
Creatine	-43579.9	1006121.8	0.966	-2077027.9	1989868.1
4-Methylvaleric acid/Hexanoic acid	11948.6	277173.2	0.966	-548239.3	572136.4
Benzoic acid	73.0	2585.9	0.978	-5153.3	5299.4
Hypoxanthine	27050.3	1168188.0	0.982	-2333945.6	2388046.3
Isobutyric acid	-20580.7	1087634.9	0.985	-2218772.9	2177611.5
Oxaloacetic acid	-491.1	27357.0	0.986	-55781.6	54799.4
Glyceric acid	1163.1	94844.7	0.990	-190525.1	192851.3
Taurine	-360.0	153320.6	0.998	-310232.4	309512.5

*Age, sex, BMI, and MVPA were controlled for as covariates.

Table S4 Significantly enriched enzymes as predicted by 912 metabolite sets.

Enzyme	Enrichment Ratio*	*p*
3,4-Dihydroxyl-L-pheylanaline exchange	8.0	0.003
Hydrogen-peroxide reductase 3	8.0	0.003
Triiodothyronine exchange	8.0	0.003
Tyramine O-sulfate exchange	8.0	0.003
Tyramine O-sulfate transport	8.0	0.003
Tyramine sulfotransferase	8.0	0.003
Hydrogen-peroxide oxidoreductase	8.0	0.003
Hydrogen-peroxide oxidoreductase 2	8.0	0.003
NADP+ oxidoreductase	8.0	0.003
4-Hydroxyohenylacetate exchange	8.0	0.003
Hydroxyphenylacetate transport via diffusion	8.0	0.003
Oxygen oxidoreductase	8.0	0.003
L-tyrosine carboxy-lyase	8.0	0.003
Dopa oxidase	8.0	0.003
Hydrogen-peroxide oxidoreductase 4	8.0	0.003
L-Thyroxine exchange	8.0	0.003
Tetrahydrobiopterin-4a-carbinolamine dehydratase	8.0	0.003
Enolase	6.0	0.020
Pyruvate kinase	6.0	0.020
Extracellular chitinase	4.0	0.040
N-acetyl-D-glucosamine exchange	4.0	0.040
Peroxisomal FAD transporter	4.0	0.045

*Analyzed as high screen time/low screen time

Table S5 Taxa with greatest metabolite cooccurrence as produced by mmvec analysis.

Taxa	Metabolite	mmvec Rank
k__Bacteria;p__Actinobacteria;c__Coriobacteriia;o__Coriobacteriales;f__Coriobacteriaceae;g__Collinsella	Isoleucine	4.11
	L-Alloisoleucine/Leucine/Norleucine	3.71
	Valine	3.51
	Phenylalanine	3.10
	Proline	3.10
k__Bacteria;p__Firmicutes;c__Bacilli;o__Lactobacillales;__;__	Isoleucine	4.44
	L-Alloisoleucine/Leucine/Norleucine	3.99
	Valine	3.72
	Proline	3.48
	Phenylalanine	3.28
k__Bacteria;p__Firmicutes;c__Clostridia;o__Clostridiales;f__Lachnospiraceae;g__Ruminococcus	Isoleucine	6.58
	L-Alloisoleucine/Leucine/Norleucine	6.18
	Proline	5.67
	Valine	5.38
	Phenylalanine	4.91
k__Bacteria;p__Firmicutes;c__Erysipelotrichi;o__Erysipelotrichales;f__Erysipelotrichaceae;g__cc_115	Isoleucine	4.56
	L-Alloisoleucine/Leucine/Norleucine	4.06
	Valine	3.79
	Proline	3.65
	Phenylalanine	3.33
k__Bacteria;p__Firmicutes;c__Bacilli;o__Turicibacterales;f__Turicibacteraceae;g__Turicibacter	Isoleucine	4.49
	L-Alloisoleucine/Leucine/Norleucine	4.01
	Valine	3.69
	Proline	3.54
	Phenylalanine	3.24

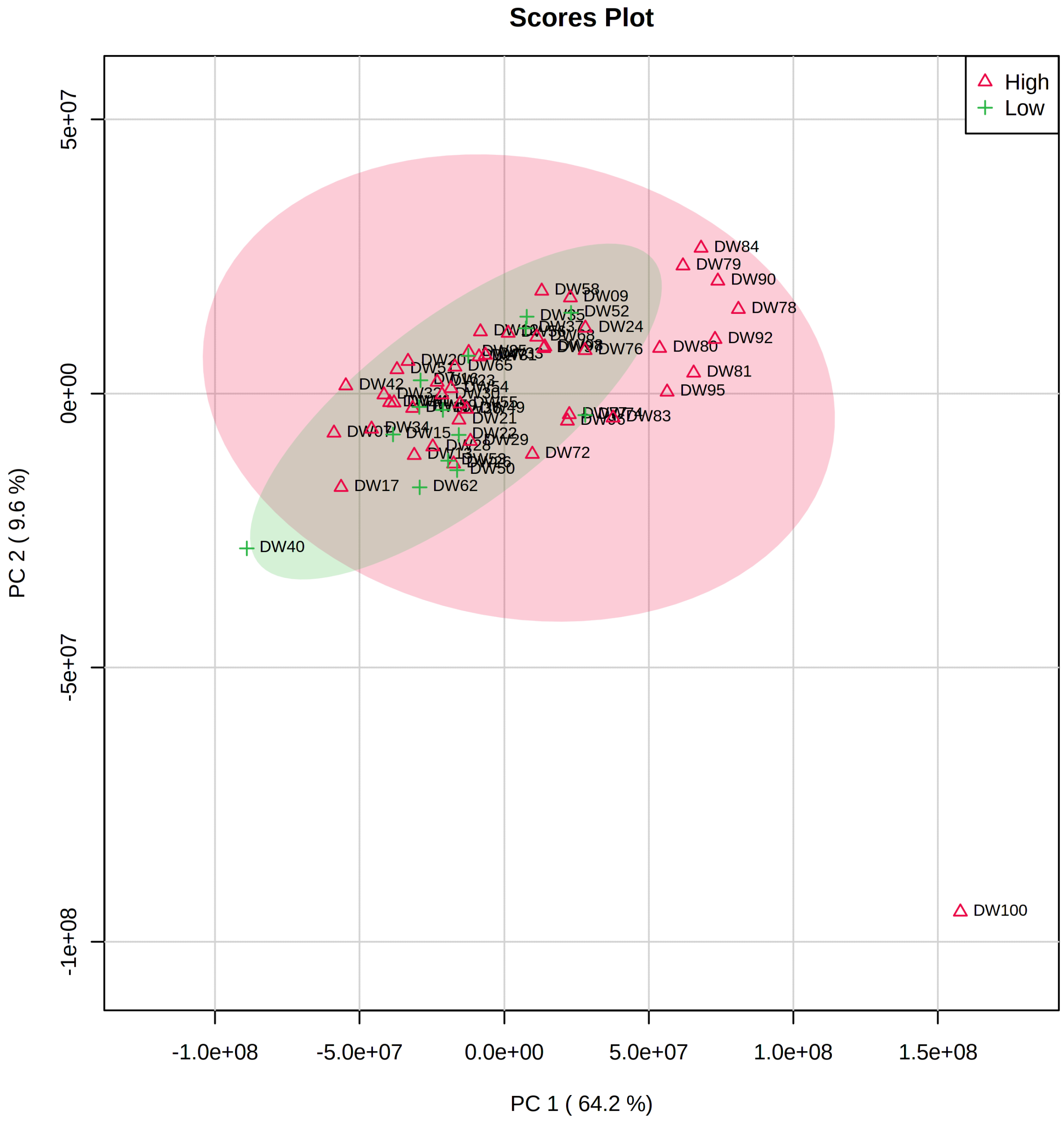

Fig. S1 Two dimensional scores plot of PCA conducted using the entire set of captured metabolites and GM features between groups. Analysis shows two potential outliers (DW40 and DW100) that were removed prior to additional analyses.

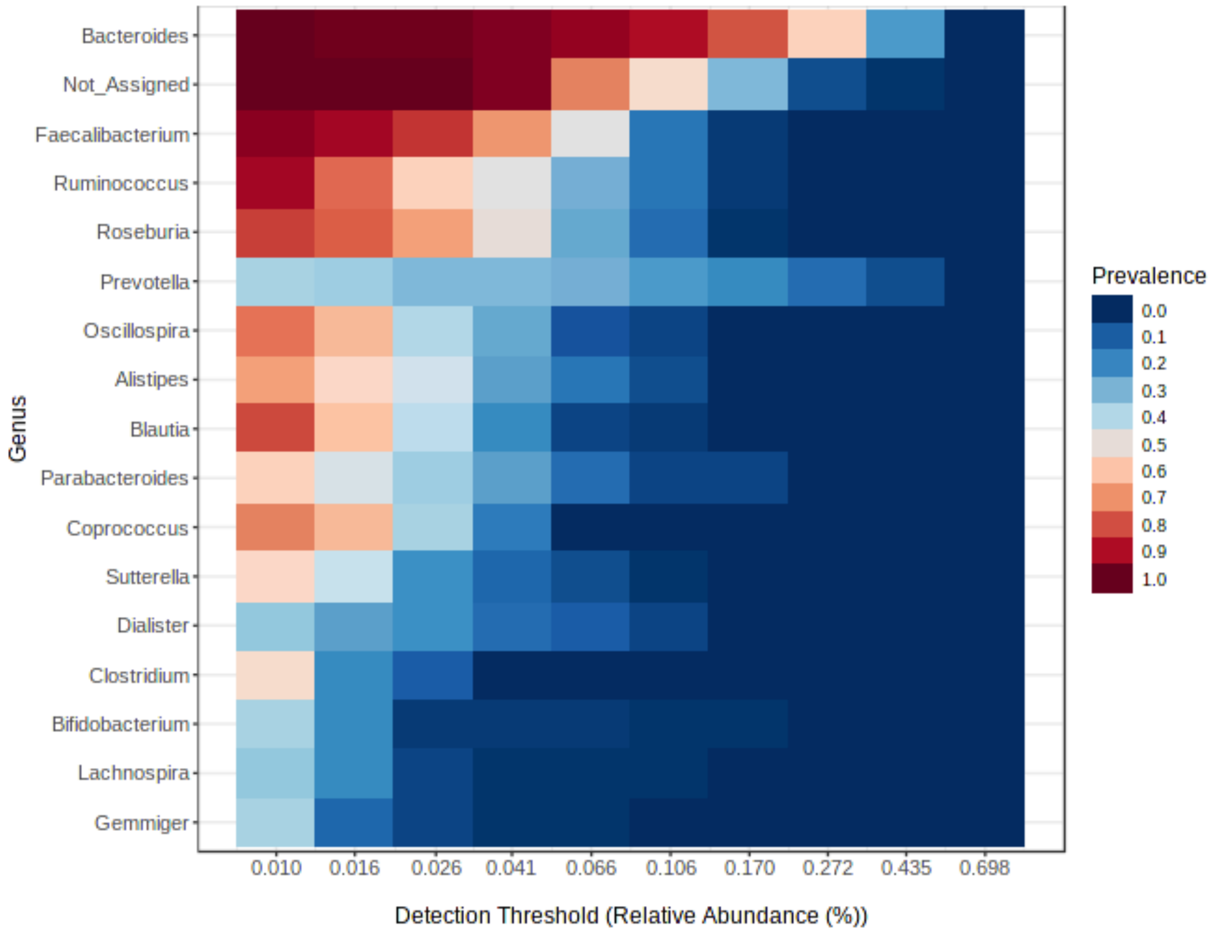

Fig. S2 Heatmap of core microbiome. Results are displayed as prevalence by detection threshold at the genus level.

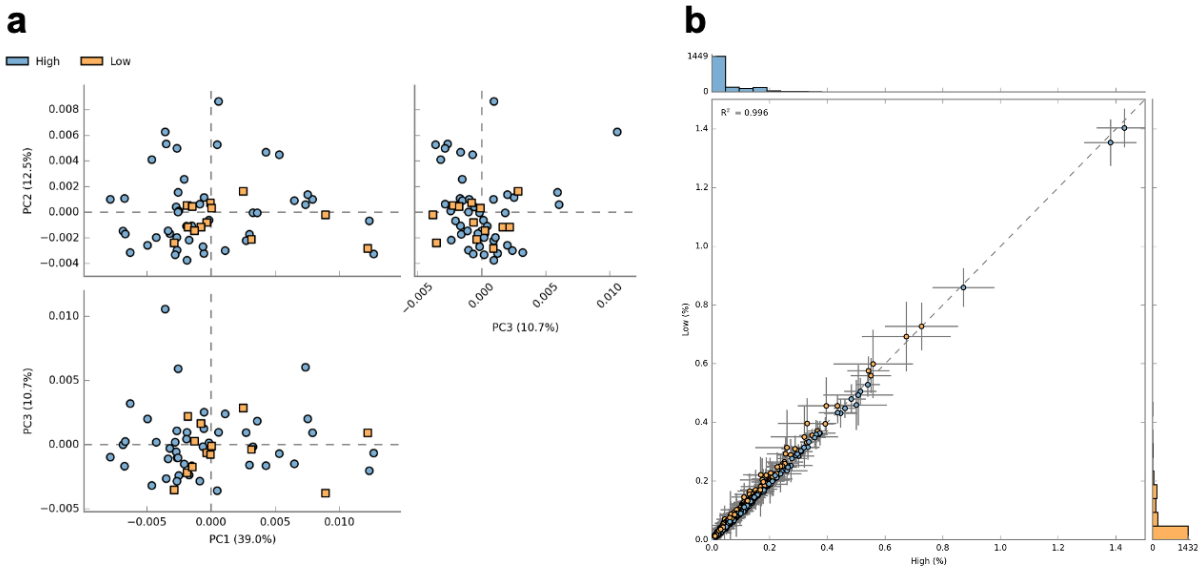

Fig. S3 Predicted functional metabolic pathways by high vs low screen time. **a** PCA analysis. **b** Scatter plot displaying the predicted functional differences between high vs low screen time. Gray lines indicate 95% CIs for each feature.

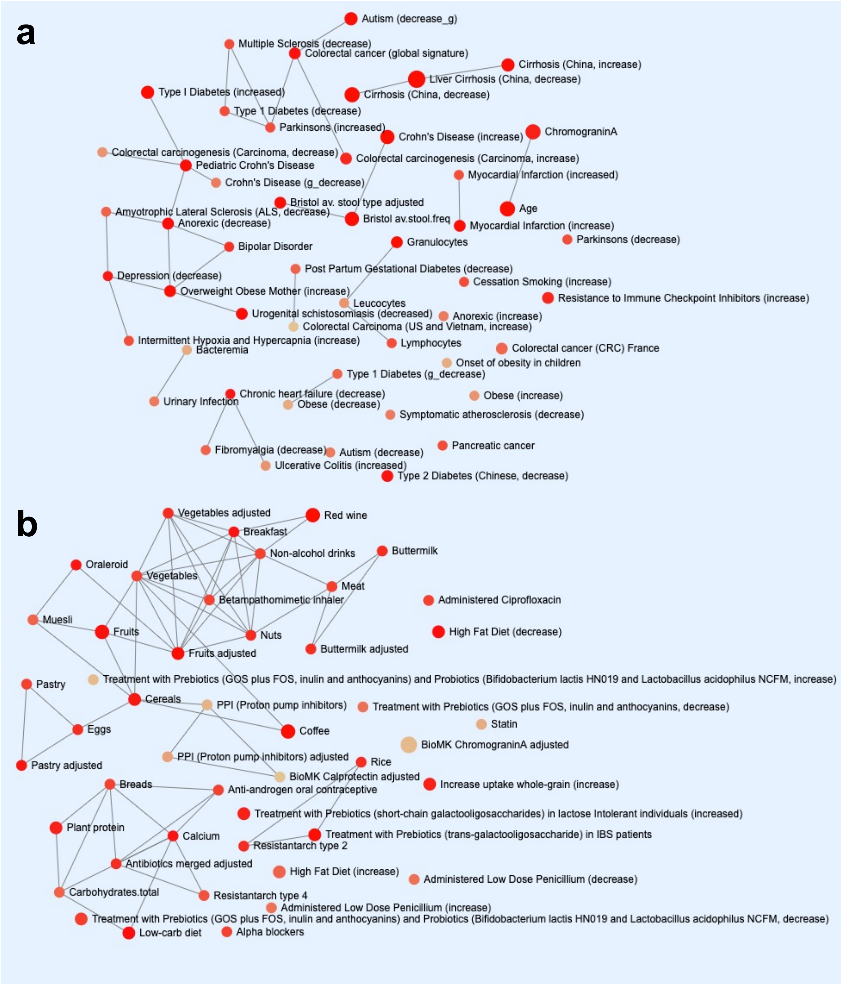

Fig. S4 TSEA of microbiome data. **a** Network view of 239 taxon sets associated with host-intrinsic factors such as disease. Significant taxon sets (*q*): liver cirrhosis (0.001), Crohn's disease (0.001), type I diabetes (0.003) overweight/obese mother (0.003), autism (0.006), myocardial infarction (0.008). Liver cirrhosis and autism were predicted to be decreased in high screen time group; Crohn's disease, type I diabetes, overweight/obese mother, and myocardial infarction were predicted to be increased in high screen time group. **b** Network view of 118 taxon sets associated with host-extrinsic factors including diet and lifestyle. Significant taxon sets (*q*): red wine (0.007), coffee (0.025), high fat diet (0.047). Consumption of a high-fat diet was predicted to be decreased in high screen time group; consumption of red wine and coffee were predicted to be increased in high screen time group.

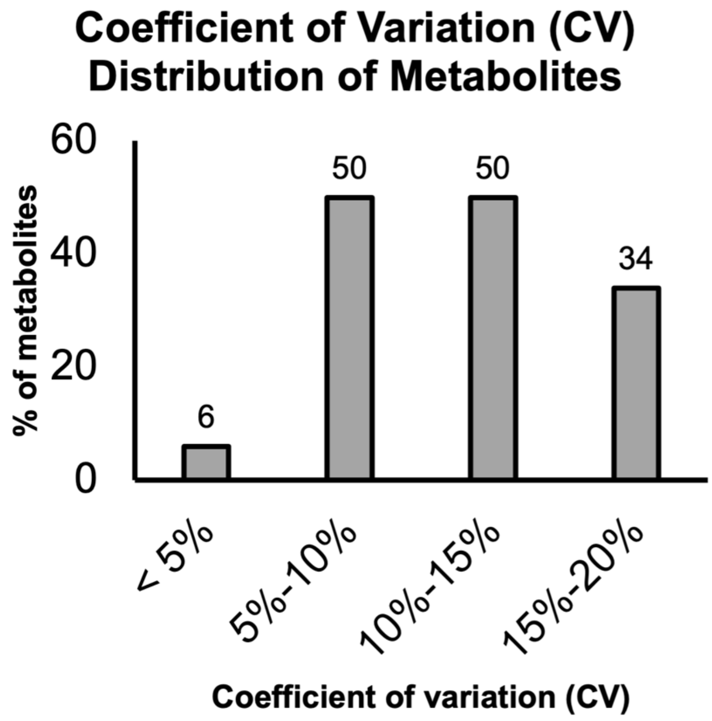

Fig. S5 Distribution of CV values of all measured metabolites in this study. QC CV range: 1.25%-20.0%, median CV: 11.5%, with ~76% of metabolites showing CV < 15%.

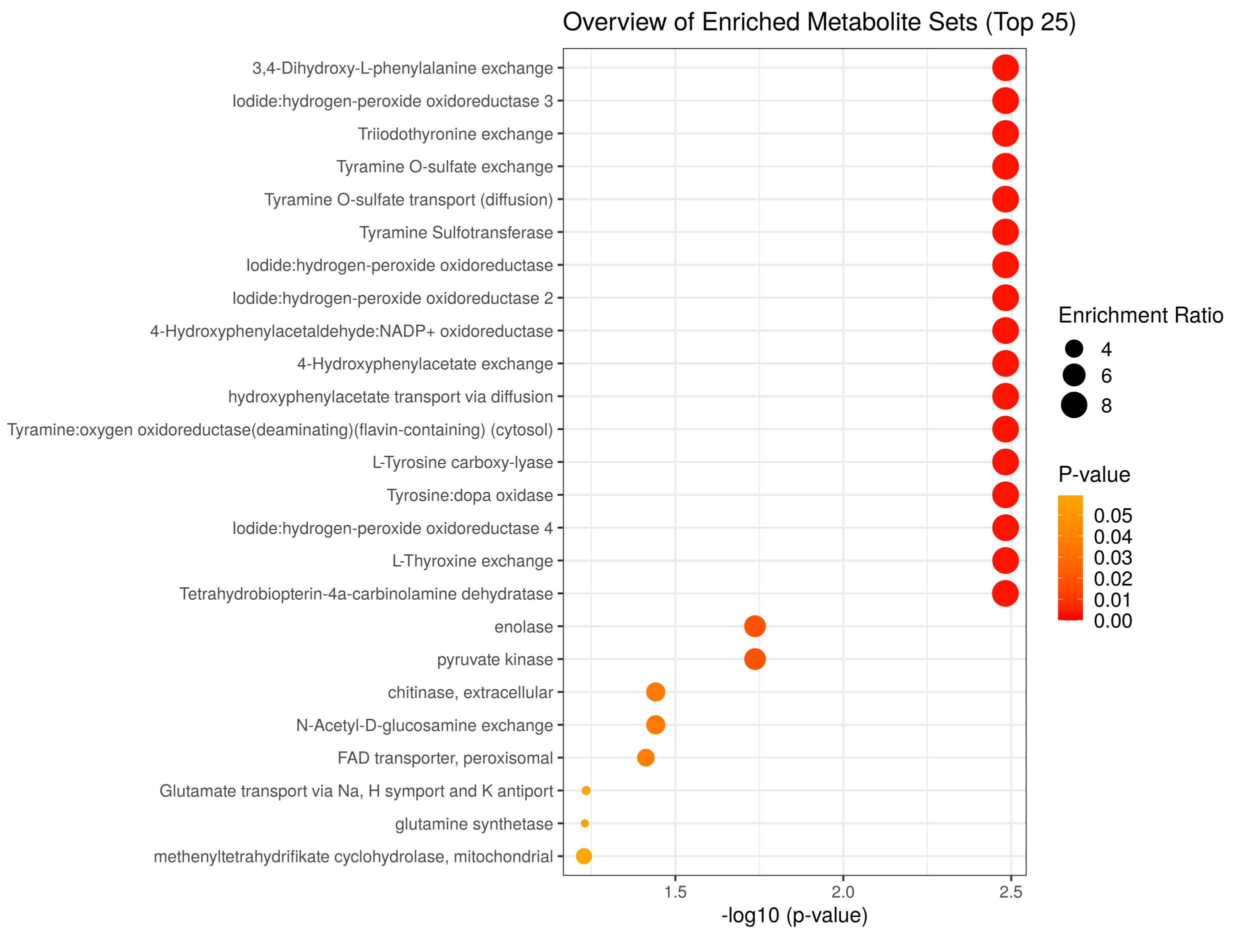

Fig. S6 Enzyme enrichment analysis of metabolomic data performed using 912 metabolic sets predicted to change in the case of dysfunctional enzymes.

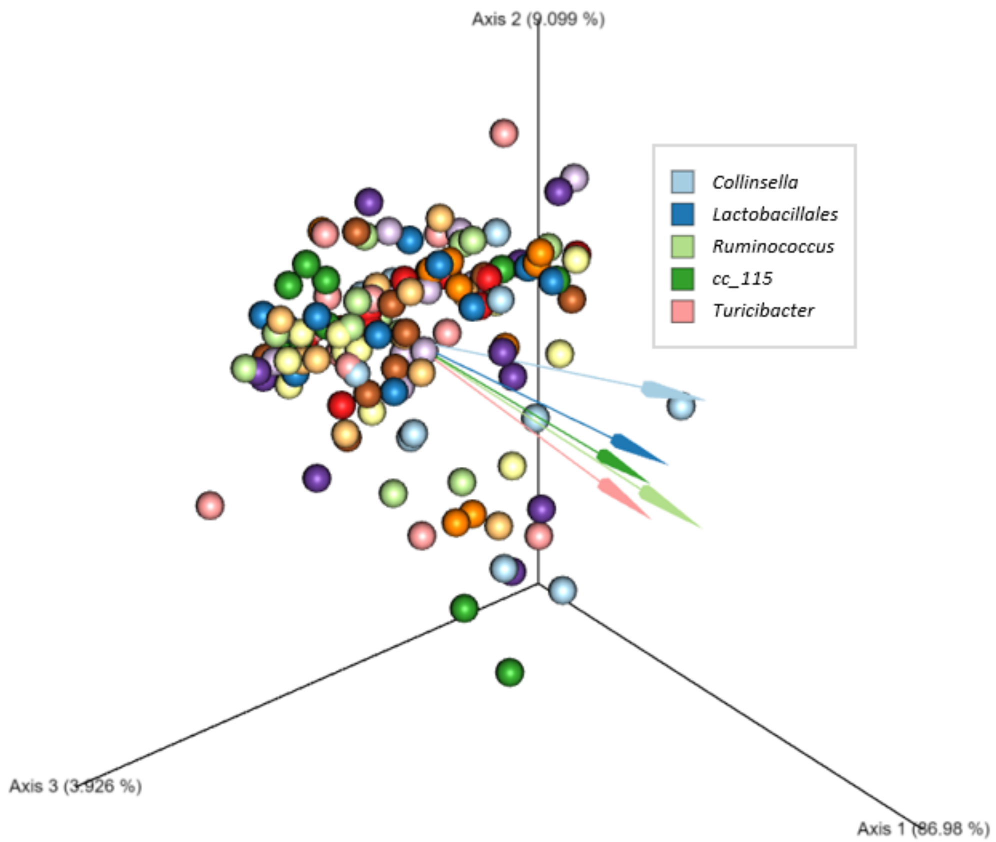

Fig. S7 Integrative analysis of 16S sequencing and metabolomic data sets. Taxa are represented by arrows indicating their co-occurrences with metabolites (dots).
